# Role of the cAMP-Dependent Carbon Catabolite Repression in Capsular Polysaccharide Biosynthesis in *Klebsiella pneumoniae*

**DOI:** 10.1371/journal.pone.0054430

**Published:** 2013-02-11

**Authors:** Ching-Ting Lin, Yu-Ching Chen, Tzyy-Rong Jinn, Chien-Chen Wu, Yi-Ming Hong, Wen-Hao Wu

**Affiliations:** 1 School of Chinese Medicine, China Medical University, Taichung, Taiwan. Republic of China; 2 Department of Biomedical Informatics, Asia University, Taichung, Taiwan. Republic of China; 3 Department of Biological Science and Technology, National Chiao Tung University, Hsin Chu, Taiwan, Republic of China; University Medical Center Utrecht, The Netherlands

## Abstract

*K. pneumoniae* is the predominant pathogen isolated from liver abscesses of diabetic patients in Asian countries. Although elevated blood glucose levels cause various immune problems, its effects on *K. pneumoniae* virulence are unknown. This study investigated the regulation of capsular polysaccharide (CPS) biosynthesis, a major determinant for *K. pneumoniae* virulence, in response to exogenous glucose. We found that *K. pneumoniae* produce more CPS in glucose-rich medium via reduction in cyclic AMP (cAMP) levels. Individual deletion of *cyaA* or *crp,* which respectively encode adenylate cyclase and cAMP receptor protein in *K. pneumoniae*, markedly increased CPS production, while deletion of *cpdA*, which encodes cAMP phosphodiesterase, decreased CPS production. These results indicate that *K. pneumoniae* CPS biosynthesis is controlled by the cAMP-dependent carbon catabolite repression (CCR). To investigate the underlying mechanism, quantitative real-time PCR and promoter-reporter assays were used to verify that the transcription of CPS biosynthesis genes, which are organized into 3 transcription units (*orf1-2*, *orf3-15*, and *orf16-17*), were activated by the deletion of *crp*. Sequence analysis revealed putative CRP binding sites located on P*_orf3-15_* and P*_orf16-17_*, suggesting direct CRP-cAMP regulation on the promoters. These results were then confirmed by electrophoretic mobility shift assay. In addition, we found putative CRP binding sites located in the promoter region of *rcsA*, which encodes a *cps* transcriptional activator, demonstrating a direct repression of CRP-cAMP and P*_rcsA_*. The deletion of *rcsA* in mutation of *crp* partially reduced CPS biosynthesis and the transcription of *orf1-2* but not of *orf3-15* or *orf16-17*. These results suggest that RcsA participates in the CRP-cAMP regulation of *orf1-2* transcription and influences CPS biosynthesis. Finally, the effect of glucose and CCR proteins on CPS biosynthesis also reflects bacterial resistance to serum killing. We here provide evidence that *K. pneumoniae* increases CPS biosynthesis for successful infection in response to exogenous glucose via cAMP-dependent CCR.

## Introduction

*Klebsiella pneumoniae* is a Gram-negative pathogen which causes suppurative lesions, bacteremia, and urinary as well as respiratory tract infections mostly in patients with underlying diseases [1]. In Asian countries, especially in Taiwan and Korea, *K. pneumoniae* is the predominant pathogen responsible for pyogenic liver abscess in diabetic patients [2], [3], [4]. In recent years, reports of *Klebsiella* liver abscess (KLA) in western countries have also been accumulating [5]. Among the virulence factors identified in *K. pneumoniae*, capsular polysaccharide (CPS) is considered as the major determinant for *K. pneumoniae* virulence. Pyogenic liver abscess isolates often carry heavy CPS loads that could protect the bacteria from phagocytosis and killing by serum factors [6], [7]. The capsular serotypes of *K. pneumoniae* have been classified into more than 77 known types [8], [9]. In Taiwan, a high prevalence of the K1 and K2 serotypes of *K. pneumoniae* was documented in liver abscess of diabetes mellitus patients [10]. However, the exact mechanism of the tight association between *K. pneumoniae*, liver abscess, and diabetes mellitus remains unclear.

Diabetic patients have been reported to be more susceptible to infections [11], [12]. It has also been demonstrated that *K. pneumoniae* strains are more virulent in diabetic than in normal mice [13]. The increased risk of bacterial infection in diabetic patients has been studied with regard to host immune system defects [14], [15], [16]; however, the alteration of gene expression patterns of pathogenic bacteria in response to elevated blood glucose levels awaits further investigation. First studied in *Escherichia coli* but highly conserved across bacteria, the carbon catabolite repression (CCR) regulates uptake of glucose and repression of genes required for utilization of less preferred carbon sources [17], [18], [19]. The CCR is generally controlled by the second messenger cyclic AMP (cAMP), which has a fundamental role in global gene regulation [20]. Bacteria grown in glucose show inhibited cAMP production, while bacteria grown in less-preferred carbon sources produce elevated levels of cAMP [17], [19], [21]. To balance intracellular cAMP levels, the adenylate cyclase CyaA and the cAMP phosphodiesterase CpdA, are required for cAMP biosynthesis and degradation, respectively [17], [19], [22], [23]. The cellular target for cAMP signalling is the cAMP receptor protein (CRP). To regulate mRNA transcription, CRP consists of a homodimer with cAMP and exhibits DNA-binding activity to the CRP binding site (TGTGA-N6-TCACA and TGCGA-N6-TCGCA) in promoter regions [24], [25], [26], [27]. In *E. coli*, CRP-cAMP acts as a global regulator of gene expression by controlling the expression of almost 200 operons [28], [29], [30]. In addition to the regulation of carbon metabolism genes, cAMP signalling has been demonstrated to regulate the expression of various genes encoding virulence factors, such as flagella, fimbriae, protease, exotoxin, and secretion systems in bacteria [31]–[40].

Sequence analysis revealed a high similarity between CCR proteins (CyaA, CpdA, and CRP) in *E. coli* and *K. pneumoniae*, suggesting a conserved regulatory mechanism. In the previous study, CRP-cAMP has been demonstrated to regulate the expression of citrate fermentation genes in *K. pneumoniae* under fermentative conditions [41]. However, the role of CCR proteins in *K. pneumoniae* pathogenesis is large uncharacterized. In this study, we aimed to examine the effect of glucose levels and CCR proteins on the regulation of *K. pneumoniae* CPS biosynthesis. Individual strains of *K. pneumoniae* CG43, a highly virulent liver abscess isolate of the K2 serotype, in which *cyaA*, *cpdA*, and *crp* had been deleted were constructed for the assessment of CPS production, and the regulatory mechanism of cAMP-dependent CCR in *cps* transcription was analysed.

## Results

### Glucose Stimulates CPS Biosynthesis

**Figure 1:**
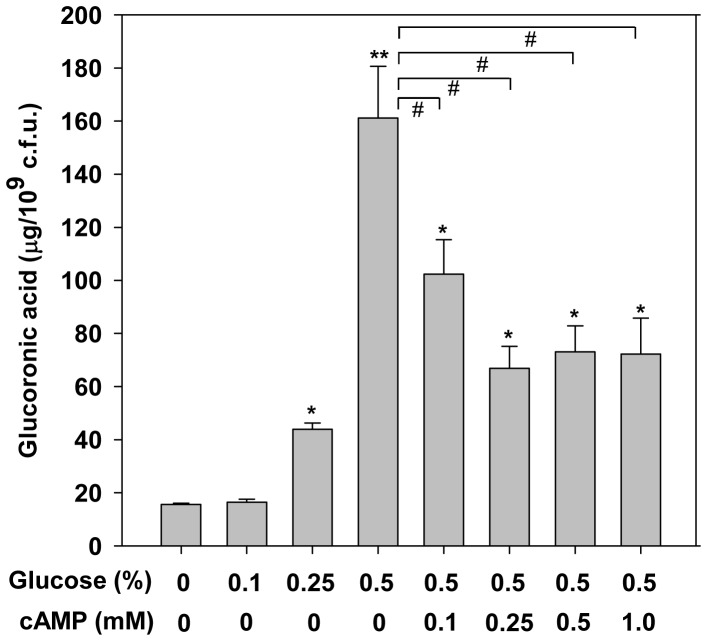
Glucose and cAMP affects the CPS levels of *K. pneumoniae* CG43S3. CPS levels of *K. pneumoniae* CG43S3 were activated by increasing environmental glucose. Bacterial strains were grown in LB broth supplemented with glucose and cAMP as indicated at 37°C with agitation. After 16 h of growth, the bacterial glucuronic acid content was determined. *P<0.05 and **P<0.01 compared with no addition. #*P*<0.05 compared to the indicated group.

To analyse if exogenous glucose affects *K. pneumoniae* CPS biosynthesis, CG43S3 was grown in LB broth supplemented with increasing amount of glucose for the quantification of CPS. As shown in Fig. 1, the CPS level increased when bacteria were grown in LB broth supplemented with 0.25% and 0.5% glucose, while the addition of 0.1% glucose did not have an obvious effect. Since the presence of glucose in the growth medium has been demonstrated to inhibit the cAMP production in many bacteria [17], [21], we examined whether the elevated CPS production was regulated by cAMP. Increasing amounts of exogenous cAMP were added to LB broth supplemented with 0.5% glucose, and bacterial CPS production was determined. The result showed that the addition of exogenous cAMP repressed the effect of glucose on CPS production, suggesting that environmental glucose can activate *K. pneumoniae* CPS biosynthesis through a reduction of cAMP level.

### CCR Proteins Affect CPS Biosynthesis

**Figure 2:**
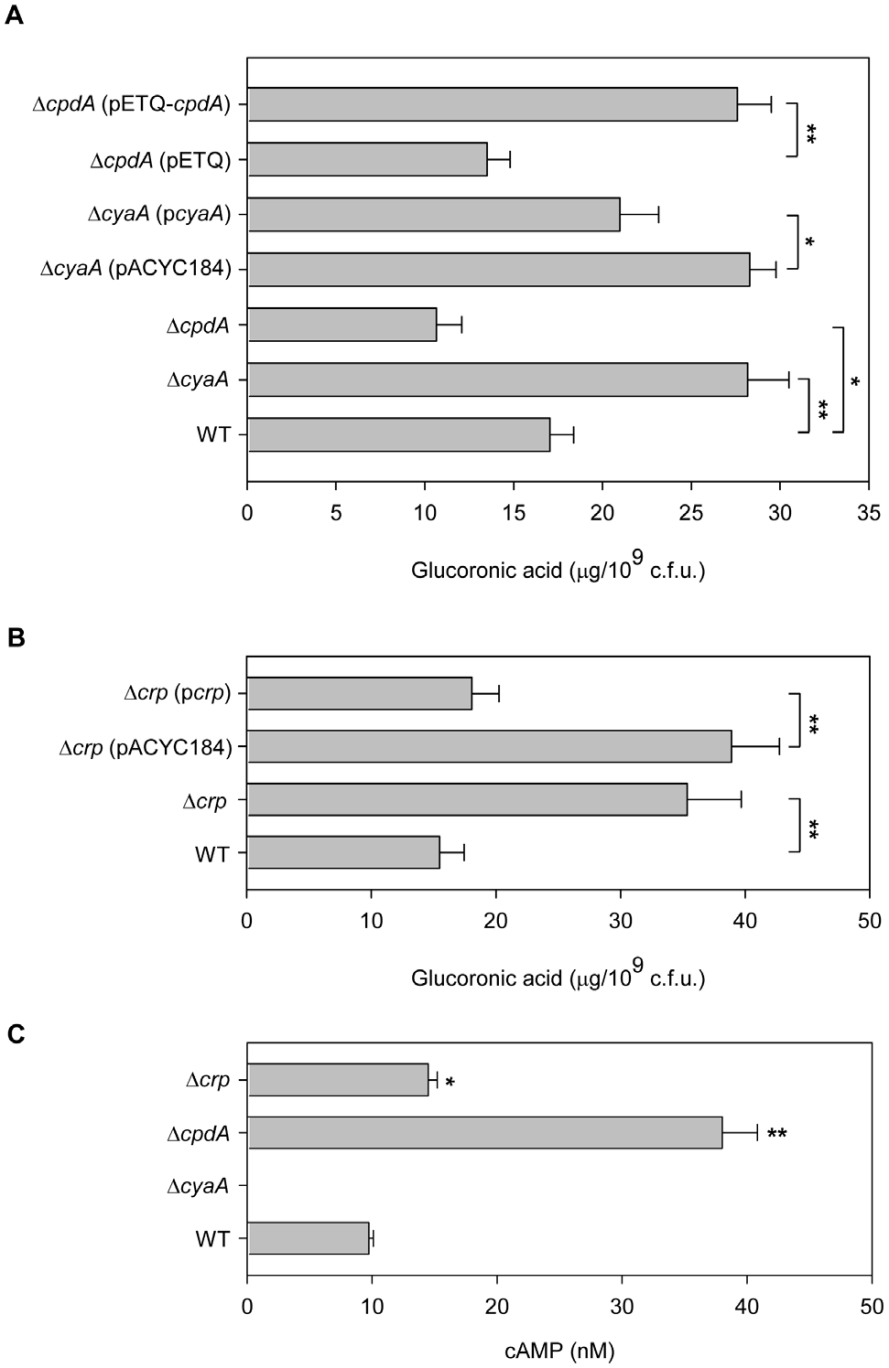
CCR proteins affect bacterial CPS levels. (A) CPS levels of WT, Δ*cyaA*, Δ*cpdA* strains and complementation of *cyaA* and *cpdA* strains were determined. (B) CPS levels in mutation and complementation of *crp* strains were determined. Bacteria were grown in LB medium at 37°C with agitation. *P<0.05 and **P<0.01 compared to the indicated group. (C) Intracellular levels of cAMP in WT, Δ*cyaA*, Δ*cpdA*, and Δ*crp* strains, as determined by ELISA. The results shown are an average from triplicate measurements in one single experiment representative of three independent experiments. Error bars indicate standard deviations. *P<0.05 and **P<0.01 compared with WT.

To confirm that *K. pneumoniae* CPS biosynthesis is regulated by cAMP, individual strains with deletion of *cyaA* and *cpdA*, which respectively encodes adenylate cyclase and cAMP phosphodiesterase from CG43S3, were constructed, and the effects of the deletions on CPS production were analysed. As shown in Fig. 2A, compared to wild type (WT) strain, we found that CPS levels increased in Δ*cyaA*, and introduction of pcyaA, but not the empty vector control (pACYC184), into Δ*cyaA* could reverse the effect of *cyaA* mutation. In contrast, the deletion of *cpdA* caused a decreased in CPS levels, which could be complemented by introducing a plasmid-carried *cpdA* (pETQ-*cpdA*) into the Δ*cpdA* strain. These results confirmed that cAMP can act as a signalling molecule for regulation of CPS biosynthesis. In addition, since cAMP affects gene transcription through its effector protein CRP, we assessed the effect of deletion of *crp* on CPS levels. As shown in Fig. 2B, compared to WT, Δ*crp* produced higher levels of CPS. Introduction of the complement plasmid p*crp*, but not the empty vector control (pACYC184), into Δ*crp* reversed the effect of the deletion. This result indicates that the CRP-cAMP signalling pathway is involved in the regulation of CPS biosynthesis, and that CRP acts as a negative regulator of CPS biosynthesis. In addition, since the functions of CyaA, CpdA, and CRP in controlling the cAMP level in *K. pneumoniae* have not yet been demonstrated, enzyme-linked immunosorbent assays were performed to determine the intracellular cAMP level upon the deletion of *cyaA*, *cpdA*, or *crp*. Compared to WT (9.75±0.35 nM), the intracellular cAMP level was almost undetectable in Δ*cyaA* (<1 nM), whereas a higher cAMP level was found in Δ*cpdA* (38±2.8 nM) (Fig. 2C). In addition, a slight increase in the cAMP level was found in Δ*crp* (14.5±0.7 nM). These result confirmed that CyaA and CpdA are responsible for cAMP biosynthesis and degradation, respectively, in *K. pneumoniae*.

### Effect of cAMP-dependent CCR on *cps* Transcription

**Figure 3:**
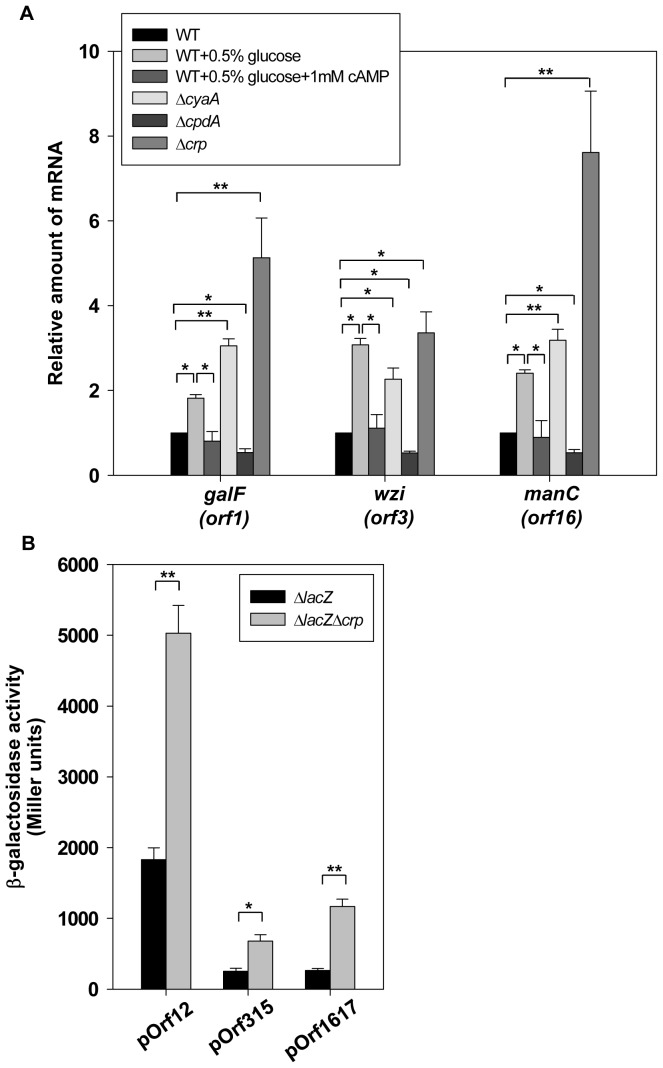
Glucose and CCR proteins affect *cps* transcription. (A) qRT-PCR analyses of the expression of the K2 *cps* genes (*orf1*, *orf3*, and *orf16*) for WT, Δ*cyaA*, Δ*cpdA*, and Δ*crp* strains in LB or indicated LB medium. (B) β-galactosidase activities of *K. pneumoniae* CG43S3Δ*lacZ* and the isogenic strain (Δ*lacZ*Δ*crp*) carrying the reporter plasmid pOrf12 (P*_orf1-2_*::*lacZ)*, pOrf315 (P*_orf3-15_*::*lacZ*), or pOrf1617 (P*_orf16-17_*::*lacZ)* were determined using log-phase cultures grown in LB medium. The results shown are an average from triplicate measurements in one single experiment representative of three independent experiments. Error bars indicate standard deviations. *P<0.05 and **P<0.01 compared to the indicated group.

The K2 *cps* gene cluster of *K. pneumoniae* Chedid contains 19 open reading frames (ORFs) organised into 3 transcription units, namely, *orf1–2*, *orf3–15*, and *orf16–17* [42]. To investigate the effect of glucose and cAMP-related proteins on the expression of the 3 *cps* gene clusters, the mRNA level of *orf1* (named *galF*), *orf3* (named *wzi*), and *orf16* (named *manC*) were measured by qRT-PCR in WT grown in LB medium containing 0.5% glucose with or without 1 mM cAMP. In addition, the effect of *cyaA*, *cpdA*, and*crp* mutation strains on the mRNA levels of *galF*, *wzi*, and *manC* were also determined. As shown in Fig. 3A, we found that the mRNA levels of *galF*, *wzi* and *manC* was increased in glucose-rich medium (LB+0.5% glucose), whereas addition of 1 mM cAMP to the glucose-rich medium could restore the *galF*, *wzi* and *manC* expression, similar to the trends observed in the WT strain. Furthermore, the mRNA levels of *galF*, *wzi*, and *manC* revealed an apparent increase in the Δ*cyaA* and Δ*crp* strains as compared to that observed in the WT strain. In contrast, a slight reduction in the mRNA level of *cps* genes was found in the Δ*cpdA* strain. This result indicates that *cps* mRNA expression is regulated by cAMP-dependent CCR, and CRP may acts a repressor of *cps* expression.

To further confirm whether CRP acts as a transcriptional repressor of the promoter activity of *galF*, *wzi*, and *manC*, the reporter plasmids pOrf12 (P*_orf1-2_*::*lacZ*), pOrf315 (P*_orf3-15_*::*lacZ*), and pOrf1617 (P*_orf16-17_*::*lacZ*), each carrying a *lacZ* reporter gene transcriptionally fused to the putative promoter region of the K2 *cps* gene cluster [43], were used to transform the *K. pneumoniae* strains CG43S3Δ*lacZ* and Δ*lacZ*Δ*crp*. The promoter activity measurements shown in Fig. 3B revealed that the deletion of *crp* in the Δ*lacZ* strain apparently increased the promoter activities of *galF*, *wzi*, and *manC*. These results verify that CRP represses *cps* expression at the transcriptional level.

### Determination of Transcriptional Start Sites on 3 Transcriptional Units in the K2 *cps* Gene Cluster

**Figure 4:**
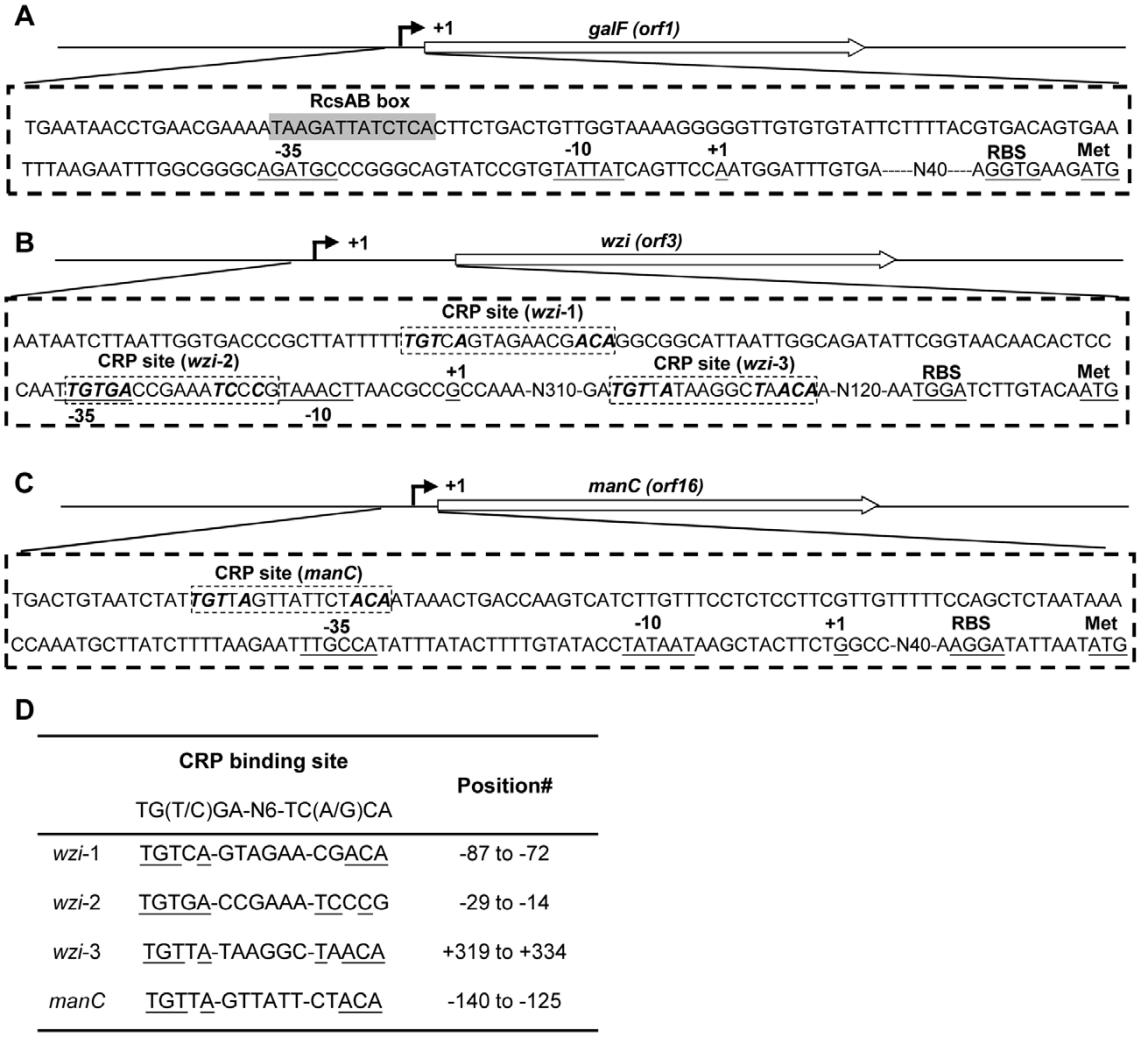
Identification of the transcriptional start sites of 3 transcriptional units in the K2 *cps* gene cluster by 5′ RACE. The 5′ RACE experimental design for *galF* (A), *wzi* (B), and *manC* (C). Relative positions of the primers used and the expected size of the PCR product are indicated. The transcriptional start site is marked as +1 and underlined. The potential −10, −35, and ribosomal binding sites (RBS) are underlined. The grey box indicates the predicted RcsAB box. The dashed boxes indicate the predicted CRP binding sites. (D) The predicted CRP binding sites in P*_wzi_* and P*_manC_* are aligned against each other. #, the position is relative to the transcriptional start site.

Until now, the transcriptional start sites of the *cps* gene cluster had not been characterized. 5′ rapid amplification of cDNA ends (5′ RACE) was first performed to determine the transcriptional start sites of the 3 transcription units in the K2 *cps* gene cluster. A single DNA band was obtained for *galF*, *wzi*, and *manC* from the 5′ RACE analysis using either primer pair (data not shown). As shown in Fig 4A, B, and C, sequence analysis of a total of 10 clones each from *galF*, *wzi*, and *manC* revealed a transcriptional start site at the A nucleotide at position −61 relative to the translational start site of *galF*, at the G nucleotide at position −470 relative to the translational start site of *wzi*, and at the G nucleotide at position −56 relative to the translational start site of *manC*. The conserved −10 and −35 promoter sequence of σ70 could be readily identified and is shown in Fig 4.

To further investigate the mechanism of CRP-cAMP regulation of *cps* gene transcription, the sequences of the *E. coli* CRP binding sites (TGTGA-N6-TCACA and TGCGA-N6-TCGCA) were used in searching the promoter sequence of the K2 *cps* gene cluster of *K. pneumoniae* Chedid. The maximum number of possible mismatched nucleotides was set at 3, and only the intergenic regions of the 3 transcriptional units were analysed in this search. Using these criteria, no typical CRP binding sites were located the upstream of *galF* (Fig. 4A). However, 3 CRP binding sites were found in the sequence upstream of *wzi* (Fig. 4B), and one CRP binding site was found to be located at position −140 to −125 relative to the transcriptional start site of *manC* (Fig. 4C and D). Among the 3 CRP binding sites were located in the intergenic regions of *orf2* and *wzi*, one was located in the 5′ mRNA region that is 137 bp upstream of the translation start site of Wzi (at position +319 to +344 relative to the transcriptional start site of *wzi*), the other 2 CRP binding sites were found to be located at position −29 to −14 and −87 to −72 relative to the transcriptional start site of *wzi* (Fig. 4D). The result implies that CRP could bind directly to the CRP binding sites that are located in P*_wzi_* and P*_manC_* for controlling *cps* expression.

### CRP Directly Binds to the CRP Binding Sites in P*_wzi_* and P*_manC_*

**Figure 5:**
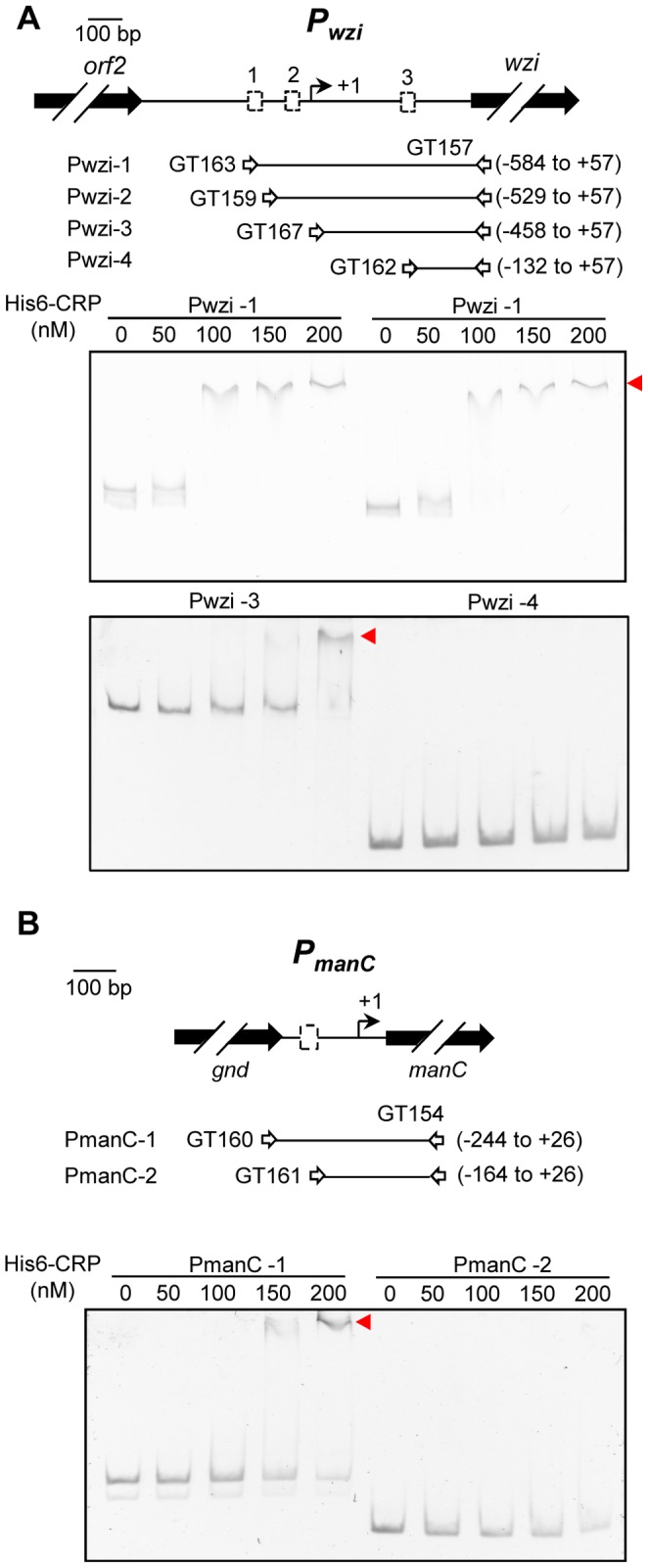
CRP directly binds to P*_wzi_* and P*_manC_*. Diagrammatic representation of the *wzi* loci (P*_orf3-15_*) (A) and the *manC* loci (P*_orf16-17_*) (B). The large arrows represent the open reading frames. The relative positions of the primer sets used in PCR-amplification of the DNA probes are indicated, and the numbers denote the positions relative to the translational start site. Names of the DNA probes are shown on the left. The dashed boxes indicate the predicted CRP consensus sequences. Different concentrations of purified His_6_-CRP were incubated with 10 ng of various truncated DNA fragments of the upstream regions of *wzi* or *manC*. Following incubation at room temperature for 30 min, the mixtures were analyzed on a 5% non-denaturing polyacrylamide gel containing 200 µM cAMP. The gel was stained with SYBR Green I dye and photographed.

To demonstrate whether CRP binds directly to the upstream sequence of *wzi* and *manC* via the CRP binding sites, electric mobility shift assay (EMSA) was performed using the recombinant His_6_-CRP protein and different DNA fragments containing truncated forms of Pwzi (Pwzi-1, Pwzi-2, Pwzi-3, and Pwzi-4) and PmanC (PmanC-1 and PmanC-2), as described in the Materials and Methods. Using Pwzi fragments of different lengths, binding of His_6_-CRP could be observed for Pwzi-1, Pwzi-2, and Pwzi-3 but not for Pwzi-4 (Fig. 5A). As shown in Fig. 5A, DNA-protein-binding complexes were observed after the incubation of 100 nM purified His_6_-CRP with 10 ng Pwzi-1 or Pwzi-2. However, formation of the Pwzi-3/CRP complex required the incubation with 200 nM purified His_6_-CRP. This suggests that the lower binding ability of His_6_-CRP in Pwzi-3 is due to the location of only one CRP binding site in Pwzi-3 as compared to 2 and 3 CRP binding sites in Pwzi-1 and Pwzi-2, respectively. In addition, we found that His_6_-CRP was able to bind to the PmanC-1 DNA fragment, but not to the PmanC-2 fragment in which the CRP binding site had been removed (Fig. 5B). This result indicates that the recombinant His_6_-CRP protein can bind directly to the predicted CRP binding sites located in P*_wzi_* and P*_manC_*.

### Expression of *rcsA* is Controlled by cAMP-dependent CCR and Directly Repressed by CRP

**Figure 6:**
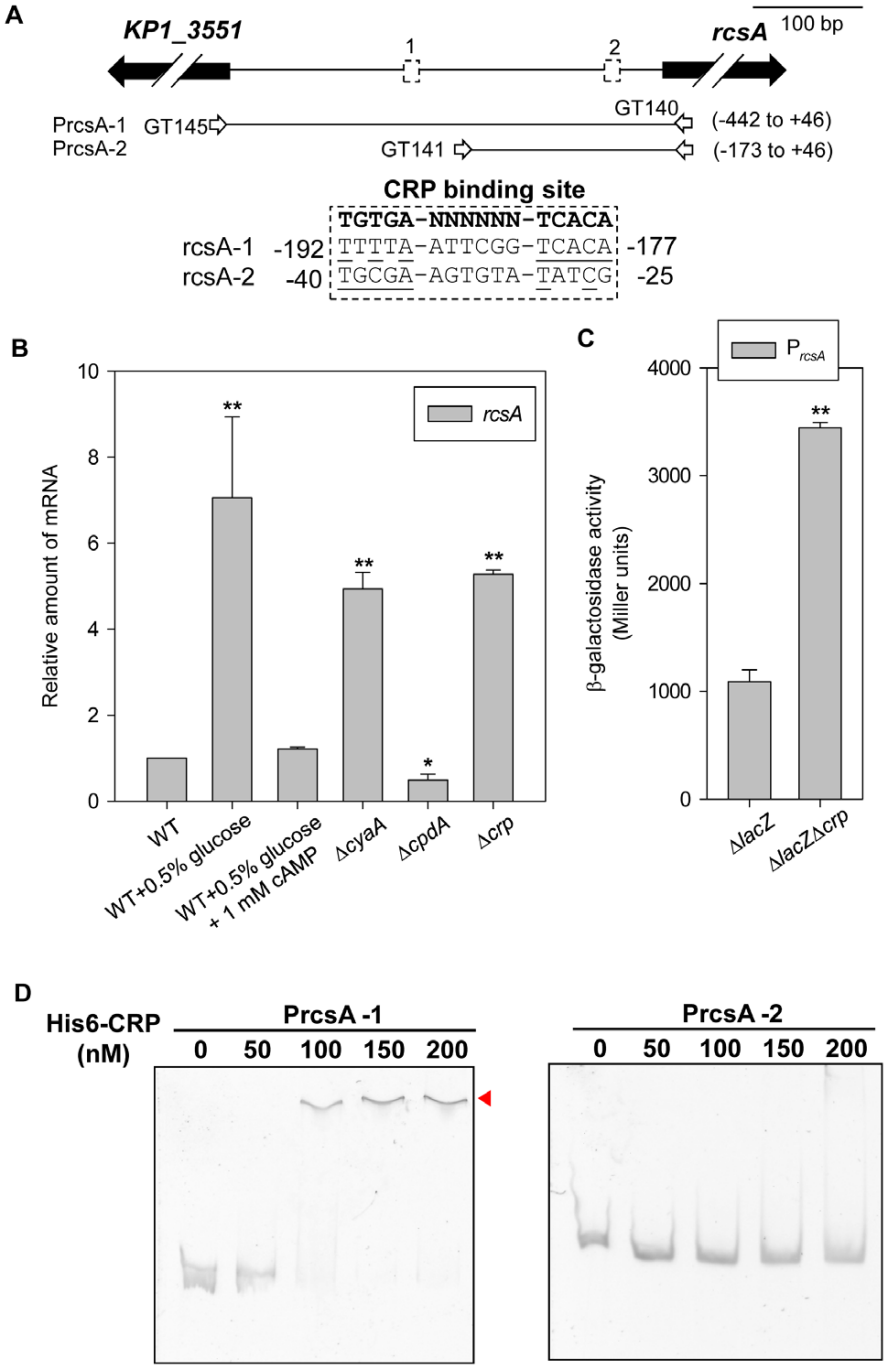
Glucose and cAMP-related proteins affect *rcsA* transcription. (A) Diagrammatic representation of *rcsA* loci. The large arrows represent the open reading frames. The relative positions of the primer sets used in PCR amplification of the DNA probes are indicated, and the numbers denote the positions relative to the translational start site. Names of the DNA probes are shown on the left. The dashed boxes indicate the predicted CRP binding sites and the alignment is shown below. (B) qRT-PCR analysis of *rcsA* expression was measured in WT, Δ*cyaA*, Δ*cpdA*, and Δ*crp* strains in LB or indicated LB medium. The results shown are an average from triplicate measurements in one single experiment representative of three independent experiments. Error bars indicate standard deviations. *P<0.05 and **P<0.01 compared with WT. (C) The β-galactosidase activities of *K. pneumoniae* CG43S3Δ*lacZ* and the isogenic strain (Δ*lacZ*Δ*crp*) carrying the reporter plasmid prcsAZ15 (P*_rcsA_*::*lacZ*) were determined using log-phased cultures grown in LB medium. **P<0.01 compared with Δ*lacZ*. (D) CRP binds directly to P*_rcsA_*. Different concentrations of purified His_6_-CRP were incubated with 10 ng of various truncated DNA fragments of the upstream region of *rcsA*. Following incubation at room temperature for 30 min, the mixtures were analyzed on a 5% non-denaturing polyacrylamide gel containing 200 µM cAMP. The gel was stained with SYBR Green I dye and photographed.

Because no CRP binding site was found in the sequence upstream of *galF*, the expression of *galF* also appeared to be controlled by cAMP-dependent CCR, implying that CRP repression of *galF* transcription is indirect and that other transcription factors are involved in the CRP regulon controlling *cps* transcription. According to previous studies, multiple regulatory proteins, which include Fur, RcsA, RcsB, RmpA, RmpA2, KvgA, and KvhR have been shown to mediate K2 *cps* expression [43], [44], [45], [46]. To further investigate whether these *cps* regulatory proteins are involved in CRP regulon, the CRP binding site was searched in the upstream sequence of *fur*, *rcsA*/*B*, *rmpA*/*A2*, *kvgA*, and *kvhR*. However, we found the 2 CRP binding sites (rcsA-1 and rcsA-2) are located at −192 to −177 and −40 to −25 relative to the translation start site of RcsA (Fig. 6A), but no typical CRP binding site was found in other upstream sequence of *fur*, *rcsB*, *rmpA*/*A2*, *kvgA*, and *kvhR*. Therefore, we suggest that RcsA is a CRP-regulated transcription factor and is involved in cAMP-dependent CCR control of *cps* expression.

To verify this possibility, the effect of glucose and cAMP-dependent CCR on the mRNA level of *rcsA* was first determined by qRT-PCR. As shown in Fig. 6B, addition of 0.5% glucose to LB medium apparently increased the mRNA level of *rcsA*, while addition of exogenous 1 mM cAMP to glucose-rich medium could restore the level of *rcsA* expression level to the same as that observed in the WT strain. In addition, the mRNA level of *rcsA* increased in the Δ*cyaA* strain, while the deletion of *cpdA* reduced on *rcsA* expression. This result indicates that *rcsA* expression is controlled by the intracellular cAMP level in response to exogenous glucose. In addition, the deletion of *crp* also increased the expression of *rcsA*, suggesting that CRP acts a transcriptional repressor of *rcsA* expression. Furthermore, measurement of promoter activity confirmed the suggestion that the deletion of *crp* caused a higher level of expression of P*_rcsA_* (Fig. 6C).

To further investigate whether CRP binds directly to the *rcsA* promoter region, EMSA was performed. As shown in Fig. 6D, DNA-protein binding complexes were observed after the incubation of 100 nM purified His_6_-CRP with 10 ng PrcsA-1, but not with PrcsA-2 in which one of the CRP binding sites was removed. Therefore, we suggested that CRP-cAMP binds directly to the CRP binding site (rcsA-1) in P*_rcsA_* to repress *rcsA* transcription.

### Role of RcsA in Regulation of CRP on CPS Biosynthesis

**Figure 7:**
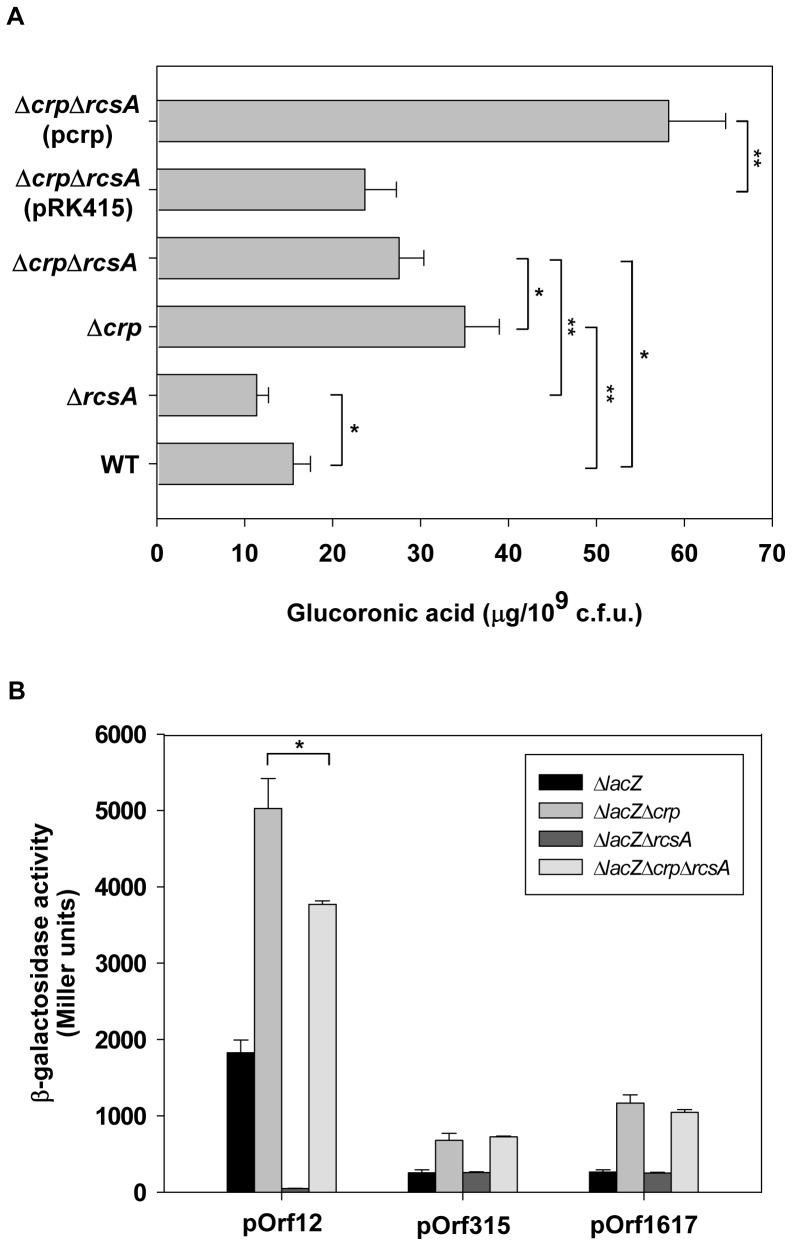
RcsA is involved in CRP regulation of CPS expression. (A) CPS levels of WT, Δ*rcsA*, Δ*crp*, and Δ*crp*Δ*rcsA* strains were determined. For complementation purposes, introduction of pRK415 and prcsA into Δ*crp*Δ*rcsA* strain were also determined. Bacterial strains were grown in LB broth as indicated at 37°C with agitation. After 16 h of growth, the bacterial glucuronic acid content was determined. *P<0.05 and **P<0.01 compared to the indicated group. (B) The β-galactosidase activities of *K. pneumoniae* CG43S3Δ*lacZ* and the isogenic strains (Δ*lacZ*Δ*crp*, Δ*lacZ*Δ*rcsA*, and Δ*lacZ*Δ*crp*Δ*rcsA*) carrying the reporter plasmid pOrf12 (P*_orf1-2_*::*lacZ)*, pOrf315 (P*_orf3-15_*::*lacZ*), or pOrf1617 (P*_orf16-17_*::*lacZ)* were determined using log-phased cultures grown in LB medium. The results shown are an average from triplicate measurements in one single experiment representative of three independent experiments. Error bars indicate standard deviations. *P<0.05 compared to the indicated group.

To understand whether RcsA participates in CRP regulation of CPS biosynthesis, the level of CPS was determined in WT, Δ*rcsA*, Δ*crp*, and Δ*crp*Δ*rcsA* strains. As shown in Fig. 7A, the deletion of *rcsA* resulted in a slight reduction in CPS level as compared to WT strain. However, the deletion of *rcsA* partially restored CPS production in the Δ*crp* strain. In addition, introducing the complementary plasmid p*rcsA* into the Δ*crp*Δ*rcsA* strain increased CPS levels as compared to the strain carrying the empty vector control (pRK415). These results indicate that RcsA participates in CRP regulation of CPS biosynthesis.

To further understand the role of RcsA in CRP regulation of *cps* transcriptions, the promoter activity of *galF* (pOrf12), *wzi* (pOrf315), and *manC* (pOrf1617) was measured in Δ*lacZ*, Δ*lacZ*Δ*crp*, Δ*lacZ*Δ*rcsA*, and Δ*lacZ*Δ*crp*Δ*rcsA* strains. As shown in Fig. 7B, the deletion of *rcsA* in Δ*lacZ* caused an apparent reduction on the promoter activity of *galF*, but no effect on the promoter activity of *wzi* and *manC* was observed, indicating that RcsA plays a positive role in *galF* transcription. In addition, the deletion of *rcsA* in the Δ*lacZ*Δ*crp* strain caused a slight reduction in the promoter activity of *galF*, unlike that in the Δ*crp* strain, but still showed higher promoter activity than the Δ*lacZ* strain. The result implies that RcsA participates in CRP regulation of *galF* transcription. However, we suggest that other unknown transcriptional regulator(s) are also involved in this regulation.

### Effect of cAMP-dependent CCR on Susceptibility to Normal Human Serum

Since CPS has been demonstrated to protect *K. pneumoniae* from killing by serum factors, we suggest that glucose, cAMP, and cAMP-related proteins may also affect the ability of *K. pneumoniae*, through modulation of CPS levels, to resist the bactericidal effects of serum. To test the hypothesis, the effects of exogenous glucose and cAMP on survival rate were first determined in treatment with 75% normal human serum. We found that the survival rate of WT grown in LB supplemented with 0.5% glucose (43.2±3.9%) increased about 2-fold as compared to the survival rate of WT in LB alone (21.2±2.3%). Addition of 1 mM cAMP diminished the higher survival rate of WT when grown in LB with 0.5% glucose (34.3±0.1%). This implies that the intracellular cAMP level decreased in *K. pneumoniae* grown in LB with 0.5% glucose, which then increased the serum resistance in *K. pneumoniae*. In addition, the deletion of *cyaA* in WT apparently increased the survival rate (40.7±0.7%), while the deletion of *cpdA* reduced the survival rate (12.7±0.6%). It also confirmed that the intracellular cAMP level could affect the ability of *K. pneumoniae* to resist the bactericidal effects of serum. Finally, the deletion of *crp* in WT had a higher survival rate (28.5±1.9%) in treatments with 75% normal human serum as compared to WT strain. Thus, the results imply that *K. pneumoniae* could resist serum killing in response to higher glucose levels via the trigger of cAMP-dependent CCR.

## Discussion

Clinically isolated *K. pneumoniae* strains usually produce a large amount of CPS, which confers not only a mucoid phenotype to the bacteria but also resistance to engulfment by phagocytes or to serum bactericidal factors [7], [47]. The degree of mucoidy has also been positively correlated with successful establishment of infection [48], [49]. Although CPS has been repeatedly proven to play an important role in *K. pneumoniae* infections and multiple CPS regulators have been found, the environmental stimuli that modulate CPS biosynthesis has remained largely unknown. Our previous studies have reported that extracellular ferric ion could repress *K. pneumoniae* CPS production through Fur regulation [44], [46]. Moreover, in the present study, we found that environmental glucose stimulated CPS production, which was regulated by cAMP (Fig. 1) and CCR proteins (Fig. 2), resulting in an increased resistance to serum killing. These findings imply that, in response to elevated blood glucose levels in diabetic patients, *K. pneumoniae* could produce more CPS to facilitate its persistence in the blood.

In *E. coli*, the expression of *cps::lacZ* was activated when cells were grown at a low temperature (20°C) in the presence of glucose (0.4%) as a carbon source [50]. As shown in Fig. 1, in *K. pneumoniae* CG43, a K2 serotype strain, we found that higher CPS production in glucose-rich medium is dependent on reducing the intracellular cAMP concentration. In addition, the activation of CPS in response to exogenous glucose was also found in *K. pneumoniae* NTUH-K2044, a highly virulent liver abscess isolate of K1 serotype, and addition of increasing amounts of exogenous cAMP in glucose-rich medium could completely reverse the effect of glucose on CPS production (Fig. S1). Therefore, we suggest that the regulatory role of glucose and cAMP-dependent CCR in CPS biosynthesis is conserved in *K. pneumoniae* stains of K1 and K2 serotype. Besides, the addition of cAMP did not completely reverse the glucose-activated CPS biosynthesis in *K. pneumoniae* CG43 (Fig. 1), suggesting that glucose could activate CPS biosynthesis through mechanism(s) other than repression of the cAMP-CRP regulation in *K. pneumoniae* CG43, which awaits further investigations.

In bacteria, CyaA and CpdA are responsible for cAMP production and degradation [22], [23]. In this study, we also found that the deletion of *cyaA* abolished the ability of cAMP to increase CPS biosynthesis, while the deletion of *cpdA* elevated the cAMP level and decreased CPS biosynthesis (Fig. 2). Although the Δ*crp* strain has a slightly higher cAMP level than WT, CPS production is obviously high in the Δ*crp* strain. This result indicated that cAMP-mediated repression of CPS biosynthesis is required for CRP regulatory activity. In *E. coli*, CRP can repress *cyaA* transcription to down-regulate cAMP production [51]. In *K. pneumoniae*, a typical CRP binding box (5′-TGTTA-AATTGA-TCACG-3′) was located at −144 to −129 relative to the translation start site of *cyaA*. In addition, the mRNA level of *cyaA* increased more than 16-fold in the Δ*crp* stain, unlike that in the WT strain (data not shown), indicating that the deletion of *crp* could increase *cyaA* expression to elevate the cAMP level in *K. pneumoniae,* consistent with the findings for *E. coli*.

To further identify the regulatory mechanism of *cps* transcription, the transcriptional start sites of 3 transcriptional units in the *cps* gene cluster were first determined (Fig. 4). CRP binds directly to the predictive CRP binding sites and represses the transcription of *wzi* and *manC* (Fig. 3 and 5), while indirectly repressing the transcription of *galF* via RcsA (Fig. 3 and 7B). According to the position of the CRP binding site relative to RNA polymerase, the CRP-activated promoter is divided into the 3 classes in *E. coli* [27], [52], [53]. However, the CRP-repressed promoter displays a greater range of binding site positions [54]. In *K. pneumoniae*, we found CRP could directly bind to the CRP binding site of *wzi-2* and *rcsA-1* centred at and near the -10 and −35 boxes, respectively, suggesting that CRP may interfere with RNA polymerase regulation of gene transcription (Fig. 4D and 6A). However, *K. pneumoniae* CRP could also repress transcription by binding directly to the predicated CRP binding site of *manC,* which was located at a relatively long distance upstream of the −10 and −35 boxes (−140 to −125) (Fig. 3 and 4D). Whether other additional transcriptional factors are required for CRP repression of *manC* transcription needs further investigation.

Multiple regulators including RcsA/B, RmpA/A2, KvhA, KvgA, and Fur have been demonstrated to control the transcription of *K. pneumoniae* CPS biosynthesis genes [43], [44], [45], [46]. In this study, we also found that *cps* transcription is regulated by cAMP-dependent CCR. In *E. coli*, CRP has been reported to regulate the expression of *fur* at the transcriptional as well as at the posttranscriptional level [55], [56], [57]. In addition, functional interaction of CRP and Fur has been demonstrated to coordinate the transcriptional regulation of iron and carbon metabolism [58]. In *K. pneumoniae*, CPS biosynthesis was modulated by iron availability and Fur has been shown to repress the transcription of *rmpA/A2* and *rcsA* to control CPS biosynthesis [46]. As shown in this study, the expression of *rcsA* is also regulated by glucose and cAMP-dependent CCR (Fig. 6). In addition, RcsA is involved in the CRP regulon in regulating *galF* expression and CPS biosynthesis (Fig. 7). By analysing the promoter sequence of *galF*, a typical RcsAB binding box [59] (5′-TAAGATTATTCTCA-3′) was found to be located at position −119 to −107 relative to the transcriptional start site of *galF*, indicating that RcsA could directly activate *galF* transcription. In addition, we noted that the deletion of *rcsA* in the Δ*lacZ*Δ*crp* strain retains a higher *galF* promoter activity compared to the Δ*lacZ* strain, implying that CRP could directly or indirectly regulate *galF* expression. Although no obvious CRP binding site was found in the promoter sequence of *galF*, an EMSA was performed to investigate whether CRP could directly bind to P*_galF_*. As shown in Fig. S2, DNA-protein binding complexes were observed after the incubation of 150 nM purified His_6_-CRP with 10 ng P*_galF_*, implying that CRP-cAMP could directly repress the *galF* transcription. However, the exact binding site of CRP in P*_galF_* needs to be further investigated. In addition, we also found that introduction of a plasmid carrying His6-CRP encoding gene into Δ*crp* strain could complement the effect of *crp* mutation on CPS biosynthesis (Fig. S3). The result confirmed that His6-CRP is functional *in vivo*. Taken together, these findings revealed a complex regulatory circuit in these CPS regulators, which then modulate the transcription of cps genes in coordination, in response to various environmental stimuli.

In *K. pneumoniae*, CPS is considered to be an important virulence factor that protects the bacteria from serum killing and phagocytosis [6], [7]. In this study, cAMP-dependent CCR was demonstrated to protect *K. pneumoniae* against serum killing, and the results suggest it plays a role in the regulation of CPS production. In addition, *E. coli* strains lacking cAMP-CRP are highly resistant to reactive oxygen species (ROS) containing hydrogen peroxide (H_2_O_2_) and hypochlorous acid (HOCl) [60]. Large amounts of ROS are generated by phagolysosomes to inhibit bacterial colonization and survival [61]. Therefore, we suggest that cAMP-dependent CCR in *K. pneumoniae* not only regulates CPS production to protect the bacteria from serum killing and phagocytosis, but also alters bacterial resistance to oxidative stress to enhance the survival rate in the phagosome, and we are currently working to demonstrate this possibility. In addition, CPS and adherence factors, such as type 1 and type 3 fimbriae, have been demonstrated to play important roles in biofilm formation and pathogenesis, and their expression could be co-regulated [62]. Biofilms are surface-attached bacteria embedded in a self-produced matrix, composed mainly of polysaccharide, but also containing proteins and nucleic acids [63]. Biofilm formation promotes encrustation and protects the bacteria from the hydrodynamic forces of urine flow, host defences and antibiotics [64]. In *E. coli* and *Serratia marcescens*, glucose/CRP-cAMP has been described to regulate the expression of type 1 fimbriae and bacterial biofilm formation [36], [37]; however, this regulation has not been proven in *K. pneumoniae*. Since glucose has also been described to repress *K. pneumoniae* biofilm formation [65], in addition to CPS, it is possible that glucose/CRP-cAMP is able to regulate the expression of adherence factors, which we are currently investigating.

In this study, we provide important evidence that glucose stimulates CPS biosynthesis in *K. pneumoniae* to protect the bacteria from serum killing, and cAMP-dependent CCR plays a profound regulatory role in CPS expression in response to glucose levels in the environment. In diabetes mellitus patients, the higher glucose level in the bloodstream is thought to have a major impact on bacterial virulence. We suggest that *K. pneumoniae* could evade the immune response via the regulation of cAMP-dependent CCR on CPS biosynthesis, especially during infection of diabetes mellitus patients. Future studies will include determining the role of cAMP-dependent CCR in modulating CPS biosynthesis, fimbria production, and biofilm formation in response to environmental stimuli.

## Materials and Methods

### Bacterial Strains, Plasmids, and Media

Bacterial strains and plasmids used in this study are listed in Table 1. Primers used in this study are list in Table 2. Bacterial were routinely cultured at 37°C in Luria-Bertani (LB) medium supplemented with appropriate antibiotics. The antibiotics used include ampicillin (100 µg/ml), kanamycin (25 µg/ml), streptomycin (500 µg/ml), and tetracycline (12.5 µg/ml).

**Table 1:** Bacterial strains and plasmids used in this study.

Strains or plasmids	Descriptions	Reference or source
*K. pneumoniae*		
CG43S3	CG43 Sm^r^, K2 serotype	[71]
NTUH-K204444	K1 serotype	From Dr. Jin-Town Wang
Δ*cyaA*	CG43S3Δ*cyaA*	This study
Δ*cpdA*	CG43S3Δ*cpdA*	This study
Δ*crp*	CG43S3Δ*crp*	This study
Δ*rcsA*	CG43S3Δ*rcsA*	[46]
Δ*crp*Δ*rcsA*	CG43S3Δ*crp*Δ*rcsA*	This study
Δ*lacZ*	CG43S3Δ*lacZ*	[43]
Δ*lacZ*Δ*crp*	CG43S3Δ*lacZ*Δ*crp*	This study
Δ*lacZ*Δ*rcsA*	CG43S3Δ*lacZ*Δ*rcsA*	This study
Δ*lacZ*Δ*crp*Δ*rcsA*	CG43S3Δ*lacZ*Δ*crp*Δ*rcsA*	This study
Δ*galU*	CG43S3Δ*galU*	[45]
*E. coli*		
DH5α	*supE44*Δ*lacU169 (f80 lacZ*ΔM15)hsdR1 *recA1 endA1 gyrA96 thi-1 relA1*	[72]
BL21(DE3)	*F^-^ ompT hsdS_B_[r_B_^-^m_B_^-^]gal dcm* [DE3]	New England Biolabs
S17-1 *λ pir*	*hsdR recA pro* RP4-2 [Tc::Mu; Km::Tn*7*] [*λpir*]	[67]
Plasmids		
pKAS46	Ap^r^ Km^r^, positive selection suicide vector, *rpsL*	[66]
yT&A	Ap^r^, TA cloning vector	Yeastern
pACYC184	Tc^r^Cm^r^, low copy number cloning vector	New England Biolabs
pRK415	Tc^r^, Broad-host-range IncP cloning vector	[73]
pcrp	Cm^r^, 987-bp fragment containing the upstream and coding region of *crp* cloned into pACYC184	This study
pETQ	Km^r^, for protein expression vector containing T5 promoter	[73]
pETQ-*cpdA*	Km^r^, 875-bp fragment containing the coding region of *cpdA* cloned into pETQ	This study
placZ15	Cm^r^, promoter selection vector, *lacZ* ^+^	[43]
prcsAZ15	Cm^r^, 488-bp fragment containing the region upstream of *rcsA* cloned into placZ15	This study
pOrf12	Cm^r^, 500-bp fragment containing the region upstream of *Klebsiella K2 cps orf1-orf2* cloned into placZ15	[43]
pOrf315	Cm^r^, 900-bp fragment containing the region upstream of *Klebsiella K2 cps orf3-orf15* cloned into placZ15	[43]
pOrf1617	Cm^r^, 300-bp fragment containing the region upstream of *Klebsiella K2 cps orf16-orf17* cloned into placZ15	[43]
pET30b-CRP	Km^r^, 654-bp fragment encoding full-length CRP cloned into pET30b	This study
pcyaA04	Ap^r^Km^r^, 2.0 kb fragment containing *cyaA* and its flanking regions cloned into pKAS46	This study
pcpdA04	Ap^r^Km^r^, 2.0 kb fragment containing *cpdA* and its flanking regions cloned into pKAS46	This study
pcrp04	Ap^r^Km^r^, 2.0 kb fragment containing *crp* and its flanking regions cloned into pKAS46	This study
prcsA	Tc^r^, 1.2-kb fragment containing the upstream and coding region of *rcsA* cloned into pRK415	This study
pcyaA	Cm^r^, 2918-bp fragment containing the upstream and coding region of *cyaA* cloned into pACYC184	This study
pETQ-His_6_-*crp*	Km^r^, 804-bp fragment containing the His_6_-*crp* cloned into pETQ	This study

**Table 2:** Primers used in this study.

Primer	Sequence (5′→3′)	Enzyme cleaved	
GT131	GGATCCTTCTACCCATTTCACACGC	*Bam*HI	
GT132	AAGCTTCAATACGCCGCTTAGCAACTT	*Hin*dIII	
GT137	GGATCCCATGGTGCTTGGCAAACC	*Bam*HI	
GT140	GGATCCCAACCGGGTATAGCTG	*Bam*HI	
GT141	AGATCTCCGGTTCTTGACTTTACTTTAAG	*Bgl*II	
GT145	CATAAAGATCTACCTGTACGCA	*Bgl*II	
GT154	GGATCCAGCCATAATCACAGGAAGCAA	*Bam*HI	
GT157	GGATCCCAGGGAGGAAAGCAAA	*Bam*HI	
GT159	AGATCTGGCAGATATTCGGTAACAACAC	*Bgl*II	
GT160	AGATCTTTATATGCCGCCCGAGTC	*Bgl*II	
GT161	AGATCTTGTTTCCTCTCCTTCGTTG	*Bgl*II	
GT162	AGATCTGCTTAGGGTAAATGTACTTGCC	*Bgl*II	
GT163	AGATCTTAATTGGTGACCCGCTTAT	*Bgl*II	
GT167	GGCCTGACCAATTATTCATCC		
GT171	GTTGTTCGACAGCTTATTGAGCTGGCGC		
GT172	GTCTAGATCCGGTAGTGGAAATCCAGA	*Xba*I	
GT173	GAATTCAACCTGCCGCAGTTCTATC	*Eco*RI	
GT174	GAATTCCACGCCGAGCGAGGC	*Eco*RI	
GT175	CCCTTCGAGCATGGCGAGCTCTAAC	*Sac*I	
GT179	AATCTCTTTGATCCCGGCGGCGACG		
GT192	TCGGTTGAAGTGTAGCCGGTAACCCGG		
GT196	CGGATCCGTCATTATCGACCACTATCC	*Bam*HI	
GT197	CAAGCTTATCCGGGCCAAATCTACG	*Hin*dIII	
GT200	TCAGCGGCTCGTTCCTTTGC		
GT202	CGGATCCAAATGGTGTCCTTAGGT	*Bam*HI	
GT203	GGGATCCTTCAGAAGGCTACTGATGGC	*Bam*HI	
GT204	GTCTAGACGTGCAGGTCTTCCACTT	*Xba*I	
GT205	GGAGCTCGATTTATACCGTTTTCG	*Sac*I	
GT210	ATGCAGATAAAGGAGCGTCGC		
GT211	GGGATCCGTCGTTAAACCTAAGGACAC	*Bam*HI	
GT227	GATATCATGCACCATCATCATCATCAT	*Eco*RI	
CC348	TTACCCCGCAATTTTCCCGCAC		
CC349	CTCGAGGGTAGGGTCTGTTTGCGGTTTGCC	*XhoI*	
CC350	CTCGAGGTTGGTCGTATTTTGAAAATGCTGGAA	*XhoI*	
CC351	ATAGAGCATGTCATCCGCCAGCAC		
HY001	AAGCTTAGTGCTGGCGATTGAGTCG	*Hin*dIII	
YCC002	ACTGGATCCTGCGACCGGAATAACC	*Bam*HI	
**For qRT-PCR**	**Sequence (5′**→**3′)**	**TaqMan probes**	**Target**
RT03	CGTCATCCAGACCAAAGAGC	83	*orf1*
RT04	CCGGTTTTTCAATAAACTCGAC		
RT05	CGATGACCGGCTTTTTAATG	83	*orf3*
RT06	CTAGCGGAGATTTGGTACTGC		
RT07	CAGTCCACCTTTATTCCGATTG	67	*orf16*
RT08	AGGTACGACCCCGACTGG		
RT11	GGTAGGGGAGCGTTCTGTAA	67	23S rRNA
RT12	TCAGCATTCGCACTTCTGAT		
RT17	TCAATAGCAATTAAGCACAAAAGAA	18	*rmpA*
RT18	TTGTACCCTCCCCATTTCC		
RT19	AAATCATTACCCACAACTAACAAAAA	80	*rmpA2*
RT20	TTAGACGGCTTTTTAATTCATGG		
GT25	AAAACAGAATCAAATATGCTGCAA	158	*rcsA*
GT26	CGTTGAGATTTGCGAAGTACC		
RT108	AGCTGCTCTTCCGATCTTGA	20	*cyaA*
RT109	AGCAGCTGACGCTCTTCG		

### Detection of cAMP

Bacteria was adjusted to 1×10^7^ colony forming units (c.f.u.)/ml and washed twice in phosphate-buffered saline (PBS). Then, the bacteria were resuspended in 300 µl of 1X lysis buffer and lysated by sonication. The lysate was centrifuged briefly at 14,000 rpm for 10 min, and the supernatant was tested for cAMP levels by using cAMP XP™ Assay Kit (Cell Signaling Technology, Inc.) and according to manufacturer’s recommendations.

### Construction of the Gene-deletion Mutants and Complementation Plasmids

Specific gene deletion containing *crp*, *cyaA*, and *cpdA* was introduced into *K. pneumoniae* CG43S3 using an allelic exchange strategy as previously described respectively [45]. In brief, two approximately 1000 bp DNA fragments flanking both sides of the deleted region were cloned into the suicide vector pKAS46 [66], a suicide vector containing *rpsL*, which allows positive selection with streptomycin for vector loss. The resulting plasmid was then mobilized from *E. coli* S17-1λ*pir* [67] to *K. pneumoniae* CG43S3, *K. pneumoniae* CG43S3Δ*lacZ*, *K. pneumoniae* CG43S3Δ*rcsA* or CG43S3-derived strains, by conjugation. The transconjugants, with the plasmid integrated into the chromosome via homologous recombination, were selected with ampicillin and kanamycin on M9 agar plates. Several of the colonies were grown in LB broth supplemented with 500 µg/mL of streptomycin to log phase at 37°C and then spread onto an LB agar plate containing 500 µg/mL of streptomycin. The streptomycin-resistant and kanamycin-sensitive colonies were selected, and the deletion was verified by PCR and Southern hybridization (data not shown). The resulting *K. pneumoniae* mutants are listed in Table 1.

To obtain the complementation plasmids, DNA fragments containing the promoter and coding sequence of *crp*, *cyaA*, and *rcsA* were individually PCR-amplified with primer pairs GT131/GT132, GT196/GT197, and HY001/GT145 (Table 2) and cloned into the shuttle vector pACYC184 or pRK415 to generate pcrp, pcyaA, and prcsA, respectively. To generate the *cpdA* complement plasmid, pETQ-cpdA, DNA fragment containing the coding sequence of *cpdA* was individually PCR-amplified with primer pair GT210/211 (Table 2) and cloned into the expression vector pETQ. To generate the His-*crp* complement plasmid, pETQ-His-*crp*, DNA fragment containing N-terminal His-tag fused with the coding sequence of *crp* was individually PCR-amplified with primer pair GT132/227 (Table 2) from pET30b-CRP and cloned into the expression vector pETQ.

### Extraction and Quantification of CPS

CPS was extracted and quantified as previously described [68]. The glucuronic acid content, represents the amount of *K. pneumoniae* K2 CPS, was determined from a standard curve of glucuronic acid (Sigma-Aldrich) and expressed as micrograms per 10^9^ c.f.u. [69].

### qRT-PCR

Total RNAs were isolated from early-exponential-phase grown bacteria cells by use of the RNeasy midi-column (QIAGEN) according to the manufacturer’s instructions. RNA was DNase-treated with RNase-free DNase I (MoBioPlus) to eliminate DNA contamination. RNA of 100 ng was reverse-transcribed with the Transcriptor First Strand cDNA Synthesis Kit (Roche) using random primers. qRT-PCR was performed in a Roche LightCycler® 1.5 Instrument using LightCycler TaqMan Master (Roche). Primers and probes were designed for selected target sequences using Universal ProbeLibrary Assay Design Center (Roche-applied science) and listed in Table 2. Data were analyzed using the real time PCR software of Roche LightCycler® 1.5 Instrument. Relative gene expressions were quantified using the comparative threshold cycle 2^-ΔΔCT^ method with 23S rRNA as the endogenous reference.

### Measurement of Promoter Activity

The promoter-reporter plasmids, pRcsAZ15, pOrf12, pOrf315, and pOrf1617, were individually mobilized into *K. pneumoniae* strains by conjugation from *E. coli* S17-1 λ*pir*. The bacteria were grown to logarithmic phase in LB broth, and the β-galactosidase activity was measured as previously described [43].

### Identification of the Transcriptional Start Sites of Three Transcriptional Units in the K2 *cps* Gene Cluster

For the determination of 5′ mRNA ends in the three transcriptional units in the K2 *cps* gene cluster, a rapid amplification of PCR was performed using 5′ RACE kit (Clontech) according to the manufacturer’s instruction as previously described [70]. A total of ten clones each of *galF*, *wzi*, and *manC*, respectively, were subjected to sequence analysis, and the transcriptional start sites of *galF*, *wzi*, and *manC* were determined. All the sequencing results indicated the same nucleotide as the transcriptional start site of *galF*, *wzi*, and *manC*.

### Purification of His_6_-CRP Protein

The coding region of *crp* was PCR amplified with primer sets GT132/GT137 (Table 2) and cloned into the *Bam*HI/*Hind*III site in pET30b (Novagen, 205 Madison, Wis). The resulting plasmid pET30b-CRP was then transformed into *E. coli* BL21(DE3) (New England Biolabs), and overproduction of the recombinant protein was induced by the addition of 0.1 mM IPTG for 4 h at 37°C. The recombinant proteins were then purified from the soluble fraction of the total cell lysate by affinity chromatography using His-Bind resin (Novagen, Madison, Wis). Finally, the purified proteins were dialyzed against 1X TE buffer (20 mM Tris-HCl, pH 8.0, 1 mM EDTA) containing 10% glycerol at 4°C overnight, and the purity was determined by SDS-PAGE.

### EMSA

The DNA fragments of the putative promoter region of *orf1-2*, *orf3-15*, *orf16-17*, and *rcsA* were respectively PCR amplified by using specific primer sets (Table 2). The purified His_6_-CRP was incubated with 10 ng DNA in a 15 µl solution containing 4 mM Tris-HCl (pH 7.4), 10 mM KCl, 100 mM dithiothreitol, 200 µM cAMP, and 10 µg/ml BSA at room temperature for 30 min. The samples were then loaded onto a native gel of 5% nondenaturing polyacrylamide containing 200 µM cAMP in 0.5X TB buffer (45 mM Tris-HCl, pH 8.0, 45 mM boric acid). Gels were electrophoresed with a 20-mA current at 4°C and then stained with SYBR Green I dye (Invitrogen).

### Bacterial Survival in Serum

Normal human serum, pooled from healthy volunteers, was divided into equal volumes and stored at −70°C before use. Bacterial survival in serum was determined with minor modifications [45]. In brief, bacteria were grown in LB broth, and when growth reached mid-exponential phase, the bacteria were collected, washed twice with phosphate-buffered saline (PBS), and then adjusted to approximately 1×10^6^ c.f.u./ml. The reaction mixture containing 250 µl of the cell suspension and 750 µl of pooled human serum was incubated at 37°C for 15 min. The number of viable bacteria was then determined by plate counting. The survival rate was expressed as the number of viable bacteria treated with human serum compared to the number of pre-treatment. The assay was performed triple, each with triplicate samples. The data from one of the representative experiments are shown and expressed as the mean and standard deviation from the three samples. A CPS-deficient mutant strain, *K. pneumoniae* CG43S3Δ*galU* (*galU* encodes for an UDP-glucose pyrophosphorylase that is responsible for supplying UDP-glucose as material for CPS biosynthesis) is served as a control.

### Statistical Method

An unpaired *t*-test was used to determine the statistical significance and values of *P*<0.05 and *P*<0.01 were considered significant. The results of CPS quantification, qRT-PCR analysis, and promoter activity measurement were derived from a single experiment representative of three independent experiments. Each sample was assayed in triplicate and the mean activity and standard deviation are presented.

### Ethics Statement

For isolation of normal human serum from healthy volunteers, the procedure and the respective consent documents were approved by the Ethics Committee of the China Medical University Hospital, Taichung, Taiwan. All healthy volunteers provided written informed consent.

## Supporting Information

Figure S1 **Glucose and cAMP affects the CPS levels of** ***K. pneumoniae*** **NTUH-K2044.** CPS levels of *K. pneumoniae* NTUH-K2044 were activated by increasing environmental glucose. Bacterial strains were grown in LB broth supplemented with glucose and cAMP as indicated at 37°C with agitation. After 16 h of growth, the bacterial glucuronic acid content was determined. *P<0.05 and **P<0.01 compared with no addition. #*P*<0.05 and $ *P*<0.01 compared to the indicated group.
(TIF)

Figure S2 **CRP directly binds to P** ***_galF_*** **.** Diagrammatic representation of the *galF* loci. The large arrows represent the open reading frames. The relative positions of the primer set used in PCR-amplification of the DNA probes are indicated, and the numbers denote the positions relative to the translational start site. Name of the DNA probes are shown on the left. Different concentrations of purified His_6_-CRP were incubated with 10 ng of the upstream regions of *galF*. Following incubation at room temperature for 30 min, the mixtures were analyzed on a 5% non-denaturing polyacrylamide gel containing 200 µM cAMP. The gel was stained with SYBR Green I dye and photographed.
(TIF)

Figure S3 **Induced expression of His_6_-CRP complements the effect of** ***crp*** **mutation on CPS biosynthesis.** CPS levels of *K. pneumoniae* strains carrying the IPTG inducible vector pETQ or pETQ-His*-crp*, as shown in the left panel, were determined. Bacteria were grown in LB medium with 100 µM IPTG at 37°C with agitation. **P<0.01 compared to the indicated group.
(TIF)

## References

[1] Podschun R, Ullmann U (1998) *Klebsiella* spp. as nosocomial pathogens: epidemiology, taxonomy, typing methods, and pathogenicity factors. Clin Microbiol Rev 11: 589–603. 9767057 10.1128/cmr.11.4.589 PMC88898

[2] Han SH (1995) Review of hepatic abscess from *Klebsiella pneumoniae*. An association with diabetes mellitus and septic endophthalmitis. West J Med 162: 220–224. 7725704 PMC1022703

[3] Lau YJ, Hu BS, Wu WL, Lin YH, Chang HY, et al (2000) Identification of a major cluster of *Klebsiella pneumoniae* isolates from patients with liver abscess in Taiwan. J Clin Microbiol 38: 412–414. 10618128 10.1128/jcm.38.1.412-414.2000 PMC88736

[4] Yang YS, Siu LK, Yeh KM, Fung CP, Huang SJ, et al (2009) Recurrent Klebsiella pneumoniae liver abscess: clinical and microbiological characteristics. J Clin Microbiol 47: 3336–3339. 19692563 10.1128/JCM.00918-09 PMC2756928

[5] Lederman ER, Crum NF (2005) Pyogenic liver abscess with a focus on *Klebsiella pneumoniae* as a primary pathogen: an emerging disease with unique clinical characteristics. Am J Gastroenterol 100: 322–331. 15667489 10.1111/j.1572-0241.2005.40310.x

[6] Sahly H, Podschun R, Oelschlaeger TA, Greiwe M, Parolis H, et al (2000) Capsule impedes adhesion to and invasion of epithelial cells by Klebsiella pneumoniae. Infect Immun 68: 6744–6749. 11083790 10.1128/iai.68.12.6744-6749.2000 PMC97775

[7] Lin JC, Chang FY, Fung CP, Xu JZ, Cheng HP, et al (2004) High prevalence of phagocytic-resistant capsular serotypes of Klebsiella pneumoniae in liver abscess. Microbes Infect 6: 1191–1198. 15488738 10.1016/j.micinf.2004.06.003

[8] Fung CP, Hu BS, Chang FY, Lee SC, Kuo BI, et al (2000) A 5-year study of the seroepidemiology of Klebsiella pneumoniae: high prevalence of capsular serotype K1 in Taiwan and implication for vaccine efficacy. J Infect Dis 181: 2075–2079. 10837197 10.1086/315488

[9] Pan YJ, Fang HC, Yang HC, Lin TL, Hsieh PF, et al (2008) Capsular polysaccharide synthesis regions in Klebsiella pneumoniae serotype K57 and a new capsular serotype. J Clin Microbiol 46: 2231–2240. 18508935 10.1128/JCM.01716-07 PMC2446917

[10] Fung CP, Chang FY, Lee SC, Hu BS, Kuo BI, et al (2002) A global emerging disease of Klebsiella pneumoniae liver abscess: is serotype K1 an important factor for complicated endophthalmitis? Gut 50: 420–424. 11839725 10.1136/gut.50.3.420 PMC1773126

[11] Geerlings SE, Stolk RP, Camps MJ, Netten PM, Hoekstra JB, et al (2000) Asymptomatic bacteriuria can be considered a diabetic complication in women with diabetes mellitus. Adv Exp Med Biol 485: 309–314. 11109121 10.1007/0-306-46840-9_41

[12] Patterson JE, Andriole VT (1997) Bacterial urinary tract infections in diabetes. Infect Dis Clin North Am 11: 735–750. 9378933 10.1016/s0891-5520(05)70383-4

[13] Wu JH, Tsai CG (2005) Infectivity of hepatic strain *Klebsiella pneumoniae* in diabetic mice. Exp Biol Med (Maywood) 230: 757–761. 16246903 10.1177/153537020523001009

[14] Muller LM, Gorter KJ, Hak E, Goudzwaard WL, Schellevis FG, et al (2005) Increased risk of common infections in patients with type 1 and type 2 diabetes mellitus. Clin Infect Dis 41: 281–288. 16007521 10.1086/431587

[15] Peleg AY, Weerarathna T, McCarthy JS, Davis TM (2007) Common infections in diabetes: pathogenesis, management and relationship to glycaemic control. Diabetes Metab Res Rev 23: 3–13. 16960917 10.1002/dmrr.682

[16] Chen SL, Jackson SL, Boyko EJ (2009) Diabetes mellitus and urinary tract infection: epidemiology, pathogenesis and proposed studies in animal models. J Urol 182: S51–56. 19846134 10.1016/j.juro.2009.07.090

[17] Botsford JL, Harman JG (1992) Cyclic AMP in prokaryotes. Microbiol Rev 56: 100–122. 1315922 10.1128/mr.56.1.100-122.1992 PMC372856

[18] Deutscher J (2008) The mechanisms of carbon catabolite repression in bacteria. Curr Opin Microbiol 11: 87–93. 18359269 10.1016/j.mib.2008.02.007

[19] McDonough KA, Rodriguez A (2011) The myriad roles of cyclic AMP in microbial pathogens: from signal to sword. Nat Rev Microbiol 10: 27–38. 22080930 10.1038/nrmicro2688 PMC3785115

[20] Saier MH Jr (1996) Cyclic AMP-independent catabolite repression in bacteria. FEMS Microbiol Lett 138: 97–103. 9026456 10.1111/j.1574-6968.1996.tb08141.x

[21] Peterkofsky A, Gazdar C (1971) Glucose and the metabolism of adenosine 3′:5′-cyclic monophosphate in *Escherichia coli* . Proc Natl Acad Sci U S A 68: 2794–2798. 4330942 10.1073/pnas.68.11.2794 PMC389527

[22] Imamura R, Yamanaka K, Ogura T, Hiraga S, Fujita N, et al (1996) Identification of the *cpdA* gene encoding cyclic 3′,5′-adenosine monophosphate phosphodiesterase in *Escherichia coli* . J Biol Chem 271: 25423–25429. 8810311 10.1074/jbc.271.41.25423

[23] Kim HS, Kim SM, Lee HJ, Park SJ, Lee KH (2009) Expression of the *cpdA* gene, encoding a 3′,5′-cyclic AMP (cAMP) phosphodiesterase, is positively regulated by the cAMP-cAMP receptor protein complex. J Bacteriol 191: 922–930. 19028903 10.1128/JB.01350-08 PMC2632089

[24] Berg OG, von Hippel PH (1988) Selection of DNA binding sites by regulatory proteins. II. The binding specificity of cyclic AMP receptor protein to recognition sites. J Mol Biol 200: 709–723. 3045325 10.1016/0022-2836(88)90482-2

[25] Harman JG (2001) Allosteric regulation of the cAMP receptor protein. Biochim Biophys Acta 1547: 1–17. 11343786 10.1016/s0167-4838(01)00187-x

[26] Cameron AD, Redfield RJ (2006) Non-canonical CRP sites control competence regulons in *Escherichia coli* and many other gamma-proteobacteria. Nucleic Acids Res 34: 6001–6014. 17068078 10.1093/nar/gkl734 PMC1635313

[27] Ebright RH (1993) Transcription activation at Class I CAP-dependent promoters. Mol Microbiol 8: 797–802. 8394979 10.1111/j.1365-2958.1993.tb01626.x

[28] Gosset G, Zhang Z, Nayyar S, Cuevas WA, Saier MH Jr (2004) Transcriptome analysis of Crp-dependent catabolite control of gene expression in *Escherichia coli* . J Bacteriol 186: 3516–3524. 15150239 10.1128/JB.186.11.3516-3524.2004 PMC415760

[29] Martinez-Antonio A, Collado-Vides J (2003) Identifying global regulators in transcriptional regulatory networks in bacteria. Curr Opin Microbiol 6: 482–489. 14572541 10.1016/j.mib.2003.09.002

[30] Zheng D, Constantinidou C, Hobman JL, Minchin SD (2004) Identification of the CRP regulon using in vitro and in vivo transcriptional profiling. Nucleic Acids Res 32: 5874–5893. 15520470 10.1093/nar/gkh908 PMC528793

[31] Baga M, Goransson M, Normark S, Uhlin BE (1985) Transcriptional activation of a *pap* pilus virulence operon from uropathogenic *Escherichia coli* . Embo J 4: 3887–3893. 2868893 10.1002/j.1460-2075.1985.tb04162.x PMC554745

[32] Lory S, Wolfgang M, Lee V, Smith R (2004) The multi-talented bacterial adenylate cyclases. Int J Med Microbiol 293: 479–482. 15149021 10.1078/1438-4221-00297

[33] Skorupski K, Taylor RK (1997) Cyclic AMP and its receptor protein negatively regulate the coordinate expression of cholera toxin and toxin-coregulated pilus in *Vibrio cholerae* . Proc Natl Acad Sci U S A 94: 265–270. 8990197 10.1073/pnas.94.1.265 PMC19310

[34] West SE, Sample AK, Runyen-Janecky LJ (1994) The *vfr* gene product, required for *Pseudomonas aeruginosa* exotoxin A and protease production, belongs to the cyclic AMP receptor protein family. J Bacteriol 176: 7532–7542. 8002577 10.1128/jb.176.24.7532-7542.1994 PMC197210

[35] Mendez M, Huang IH, Ohtani K, Grau R, Shimizu T, et al (2008) Carbon catabolite repression of type IV pilus-dependent gliding motility in the anaerobic pathogen *Clostridium perfringens* . J Bacteriol 190: 48–60. 17981974 10.1128/JB.01407-07 PMC2223757

[36] Kalivoda EJ, Stella NA, O’Dee DM, Nau GJ, Shanks RM (2008) The cyclic AMP-dependent catabolite repression system of *Serratia marcescens* mediates biofilm formation through regulation of type 1 fimbriae. Appl Environ Microbiol 74: 3461–3470. 18424546 10.1128/AEM.02733-07 PMC2423026

[37] Muller CM, Aberg A, Straseviciene J, Emody L, Uhlin BE, et al (2009) Type 1 fimbriae, a colonization factor of uropathogenic *Escherichia coli*, are controlled by the metabolic sensor CRP-cAMP. PLoS Pathog 5: e1000303. 19229313 10.1371/journal.ppat.1000303 PMC2636892

[38] Fuchs EL, Brutinel ED, Klem ER, Fehr AR, Yahr TL, et al. (2010) In vitro and in vivo Characterization of the *Pseudomonas aeruginosa* cAMP Phosphodiesterase CpdA Required for cAMP Homeostasis and Virulence Factor Regulation. J Bacteriol. 10.1128/JB.00168-10 PMC2876501 20348254

[39] Endoh T, Engel JN (2009) CbpA: a polarly localized novel cyclic AMP-binding protein in *Pseudomonas aeruginosa* . J Bacteriol 191: 7193–7205. 19801409 10.1128/JB.00970-09 PMC2786554

[40] Stella NA, Kalivoda EJ, O’Dee DM, Nau GJ, Shanks RM (2008) Catabolite repression control of flagellum production by *Serratia marcescens* . Res Microbiol 159: 562–568. 18718529 10.1016/j.resmic.2008.07.003 PMC2606049

[41] Meyer M, Dimroth P, Bott M (2001) Catabolite repression of the citrate fermentation genes in *Klebsiella pneumoniae*: evidence for involvement of the cyclic AMP receptor protein. J Bacteriol 183: 5248–5256. 11514506 10.1128/JB.183.18.5248-5256.2001 PMC95405

[42] Arakawa Y, Wacharotayankun R, Nagatsuka T, Ito H, Kato N, et al (1995) Genomic organization of the *Klebsiella pneumoniae cps* region responsible for serotype K2 capsular polysaccharide synthesis in the virulent strain Chedid. J Bacteriol 177: 1788–1796. 7896702 10.1128/jb.177.7.1788-1796.1995 PMC176807

[43] Lin CT, Huang TY, Liang WC, Peng HL (2006) Homologous response regulators KvgA, KvhA and KvhR regulate the synthesis of capsular polysaccharide in *Klebsiella pneumoniae* CG43 in a coordinated manner. J Biochem (Tokyo) 140: 429–438. 16877448 10.1093/jb/mvj168

[44] Cheng HY, Chen YS, Wu CY, Chang HY, Lai YC, et al (2010) RmpA regulation of capsular polysaccharide biosynthesis in *Klebsiella pneumoniae* CG43. J Bacteriol 192: 3144–3158. 20382770 10.1128/JB.00031-10 PMC2901686

[45] Lai YC, Peng HL, Chang HY (2003) RmpA2, an activator of capsule biosynthesis in *Klebsiella pneumoniae* CG43, regulates K2 cps gene expression at the transcriptional level. J Bacteriol 185: 788–800. 12533454 10.1128/JB.185.3.788-800.2003 PMC142793

[46] Lin CT, Wu CC, Chen YS, Lai YC, Chi C, et al (2011) Fur regulation of the capsular polysaccharide biosynthesis and iron-acquisition systems in *Klebsiella pneumoniae* CG43. Microbiology 157: 419–429. 21071493 10.1099/mic.0.044065-0

[47] Regueiro V, Campos MA, Pons J, Alberti S, Bengoechea JA (2006) The uptake of a *Klebsiella pneumoniae* capsule polysaccharide mutant triggers an inflammatory response by human airway epithelial cells. Microbiology 152: 555–566. 16436443 10.1099/mic.0.28285-0

[48] Nassif X, Honore N, Vasselon T, Cole ST, Sansonetti PJ (1989) Positive control of colanic acid synthesis in *Escherichia coli* by *rmpA* and *rmpB*, two virulence-plasmid genes of *Klebsiella pneumoniae* . Mol Microbiol 3: 1349–1359. 2693894 10.1111/j.1365-2958.1989.tb00116.x

[49] Nassif X, Sansonetti PJ (1986) Correlation of the virulence of *Klebsiella pneumoniae* K1 and K2 with the presence of a plasmid encoding aerobactin. Infect Immun 54: 603–608. 2946641 10.1128/iai.54.3.603-608.1986 PMC260211

[50] Hagiwara D, Sugiura M, Oshima T, Mori H, Aiba H, et al (2003) Genome-wide analyses revealing a signaling network of the RcsC-YojN-RcsB phosphorelay system in *Escherichia coli* . J Bacteriol 185: 5735–5746. 13129944 10.1128/JB.185.19.5735-5746.2003 PMC193970

[51] Inada T, Takahashi H, Mizuno T, Aiba H (1996) Down regulation of cAMP production by cAMP receptor protein in *Escherichia coli*: an assessment of the contributions of transcriptional and posttranscriptional control of adenylate cyclase. Mol Gen Genet 253: 198–204. 9003304 10.1007/s004380050313

[52] Lawson CL, Swigon D, Murakami KS, Darst SA, Berman HM, et al (2004) Catabolite activator protein: DNA binding and transcription activation. Curr Opin Struct Biol 14: 10–20. 15102444 10.1016/j.sbi.2004.01.012 PMC2765107

[53] Busby S, Ebright RH (1999) Transcription activation by catabolite activator protein (CAP). J Mol Biol 293: 199–213. 10550204 10.1006/jmbi.1999.3161

[54] Gama-Castro S, Jimenez-Jacinto V, Peralta-Gil M, Santos-Zavaleta A, Penaloza-Spinola MI, et al (2008) RegulonDB (version 6.0): gene regulation model of *Escherichia coli* K-12 beyond transcription, active (experimental) annotated promoters and Textpresso navigation. Nucleic Acids Res 36: D120–124. 18158297 10.1093/nar/gkm994 PMC2238961

[55] Lin HH, Hsu CC, Yang CD, Ju YW, Chen YP, et al (2011) Negative effect of glucose on *ompA* mRNA stability: a potential role of cyclic AMP in the repression of *hfq* in *Escherichia coli* . J Bacteriol 193: 5833–5840. 21840983 10.1128/JB.05359-11 PMC3187204

[56] Vecerek B, Moll I, Afonyushkin T, Kaberdin V, Blasi U (2003) Interaction of the RNA chaperone Hfq with mRNAs: direct and indirect roles of Hfq in iron metabolism of *Escherichia coli* . Mol Microbiol 50: 897–909. 14617150 10.1046/j.1365-2958.2003.03727.x

[57] De Lorenzo V, Herrero M, Giovannini F, Neilands JB (1988) Fur (ferric uptake regulation) protein and CAP (catabolite-activator protein) modulate transcription of *fur* gene in *Escherichia coli* . Eur J Biochem 173: 537–546. 2836193 10.1111/j.1432-1033.1988.tb14032.x

[58] Zhang Z, Gosset G, Barabote R, Gonzalez CS, Cuevas WA, et al (2005) Functional interactions between the carbon and iron utilization regulators, Crp and Fur, in *Escherichia coli* . J Bacteriol 187: 980–990. 15659676 10.1128/JB.187.3.980-990.2005 PMC545712

[59] Wehland M, Bernhard F (2000) The RcsAB box. Characterization of a new operator essential for the regulation of exopolysaccharide biosynthesis in enteric bacteria. J Biol Chem 275: 7013–7020. 10702265 10.1074/jbc.275.10.7013

[60] Barth E, Gora KV, Gebendorfer KM, Settele F, Jakob U, et al (2009) Interplay of cellular cAMP levels, s^S^ activity and oxidative stress resistance in *Escherichia coli* . Microbiology 155: 1680–1689. 19372151 10.1099/mic.0.026021-0 PMC2848814

[61] Slauch JM (2011) How does the oxidative burst of macrophages kill bacteria? Still an open question. Mol Microbiol 80: 580–583. 21375590 10.1111/j.1365-2958.2011.07612.x PMC3109634

[62] Wu CC, Lin CT, Cheng WY, Huang CJ, Wang ZC, et al (2012) Fur-dependent MrkHI regulation of type 3 fimbriae in *Klebsiella pneumoniae* CG43. Microbiology 158: 1045–1056. 22262101 10.1099/mic.0.053801-0

[63] Sutherland I (2001) Biofilm exopolysaccharides: a strong and sticky framework. Microbiology 147: 3–9. 11160795 10.1099/00221287-147-1-3

[64] Warren JW (2001) Catheter-associated urinary tract infections. Int J Antimicrob Agents 17: 299–303. 11295412 10.1016/s0924-8579(00)00359-9

[65] Jackson DW, Simecka JW, Romeo T (2002) Catabolite repression of *Escherichia coli* biofilm formation. J Bacteriol 184: 3406–3410. 12029060 10.1128/JB.184.12.3406-3410.2002 PMC135108

[66] Skorupski K, Taylor RK (1996) Positive selection vectors for allelic exchange. Gene 169: 47–52. 8635748 10.1016/0378-1119(95)00793-8

[67] Miller VL, Mekalanos JJ (1988) A novel suicide vector and its use in construction of insertion mutations: osmoregulation of outer membrane proteins and virulence determinants in *Vibrio cholerae* requires *toxR* . J Bacteriol 170: 2575–2583. 2836362 10.1128/jb.170.6.2575-2583.1988 PMC211174

[68] Domenico P, Schwartz S, Cunha BA (1989) Reduction of capsular polysaccharide production in *Klebsiella pneumoniae* by sodium salicylate. Infect Immun 57: 3778–3782. 2680983 10.1128/iai.57.12.3778-3782.1989 PMC259904

[69] Blumenkrantz N, Asboe-Hansen G (1973) New method for quantitative determination of uronic acids. Anal Biochem 54: 484–489. 4269305 10.1016/0003-2697(73)90377-1

[70] Cheng HY, Chen YS, Wu CY, Chang HY, Lai YC, et al. RmpA regulation of capsular polysaccharide biosynthesis in *Klebsiella pneumoniae* CG43. J Bacteriol 192: 3144–3158. 20382770 10.1128/JB.00031-10 PMC2901686

[71] Lai YC, Peng HL, Chang HY (2001) Identification of genes induced in vivo during *Klebsiella pneumoniae* CG43 infection. Infect Immun 69: 7140–7145. 11598090 10.1128/IAI.69.11.7140-7145.2001 PMC100105

[72] Hanahan D (1983) Studies on transformation of Escherichia coli with plasmids. J Mol Biol 166: 557–580. 6345791 10.1016/s0022-2836(83)80284-8

[73] Keen NT, Tamaki S, Kobayashi D, Trollinger D (1988) Improved broad-host-range plasmids for DNA cloning in gram-negative bacteria. Gene 70: 191–197. 2853689 10.1016/0378-1119(88)90117-5

