# Supplementary figures and images for: Role of the cAMP-Dependent Carbon Catabolite Repression in Capsular Polysaccharide Biosynthesis in Klebsiella pneumoniae
Source: PLoS One. 2013 Feb 11;8(2):e54430. doi: 10.1371/journal.pone.0054430 (PMC3569464; doi:10.1371/journal.pone.0054430)

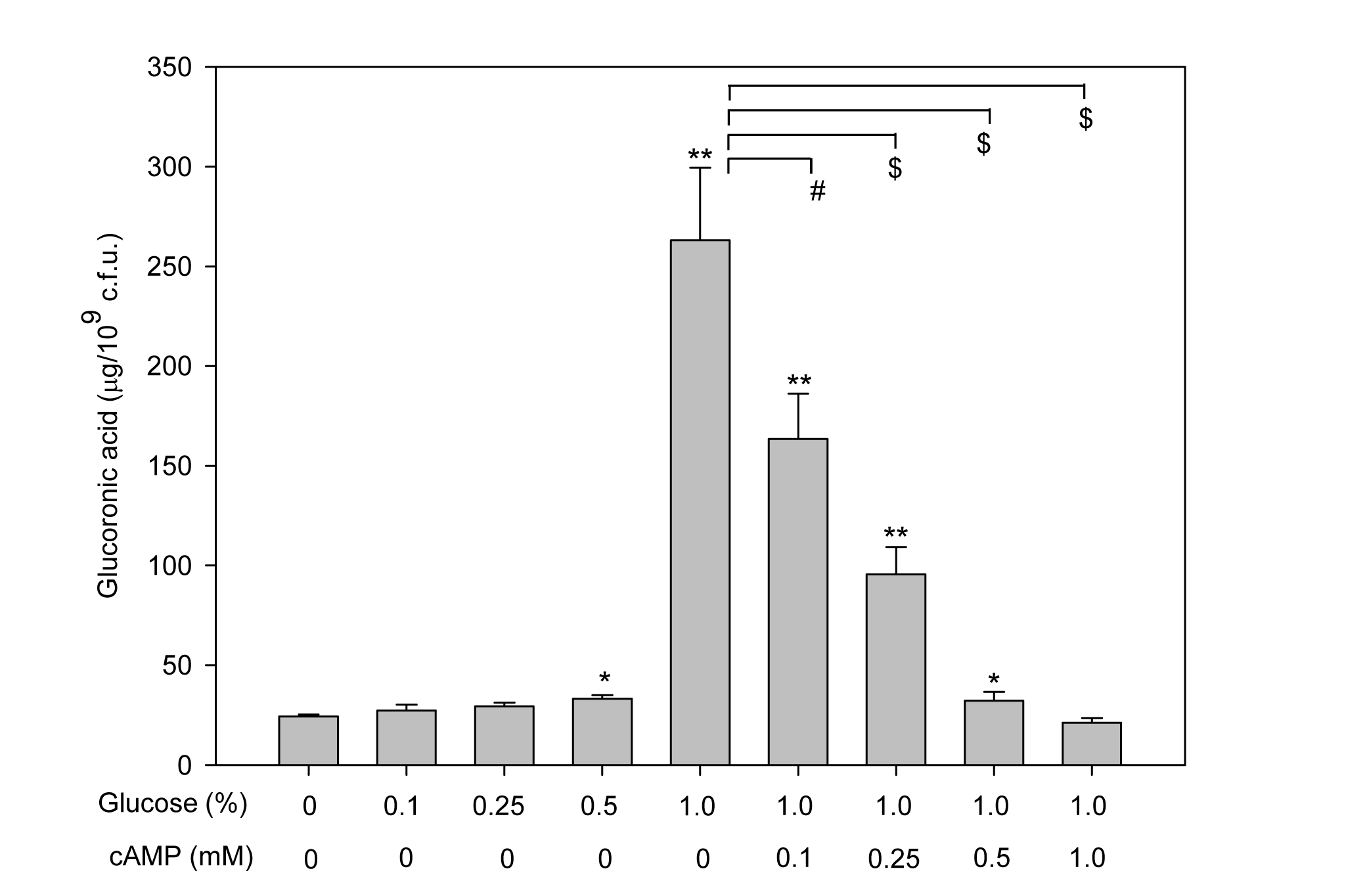

Supplement: Figure S1 — Glucose and cAMP affects the CPS levels of K. pneumoniae NTUH-K2044. CPS levels of K. pneumoniae NTUH-K2044 were activated by increasing environmental glucose. Bacterial strains were grown in LB broth supplemented with glucose and cAMP as indicated at 37°C with agitation. After 16 h of growth, the bacterial glucuronic acid content was determined. *P<0.05 and **P<0.01 compared with no addition. #P<0.05 and $ P<0.01 compared to the indicated group. (TIF) [file pone.0054430.s001.tif]

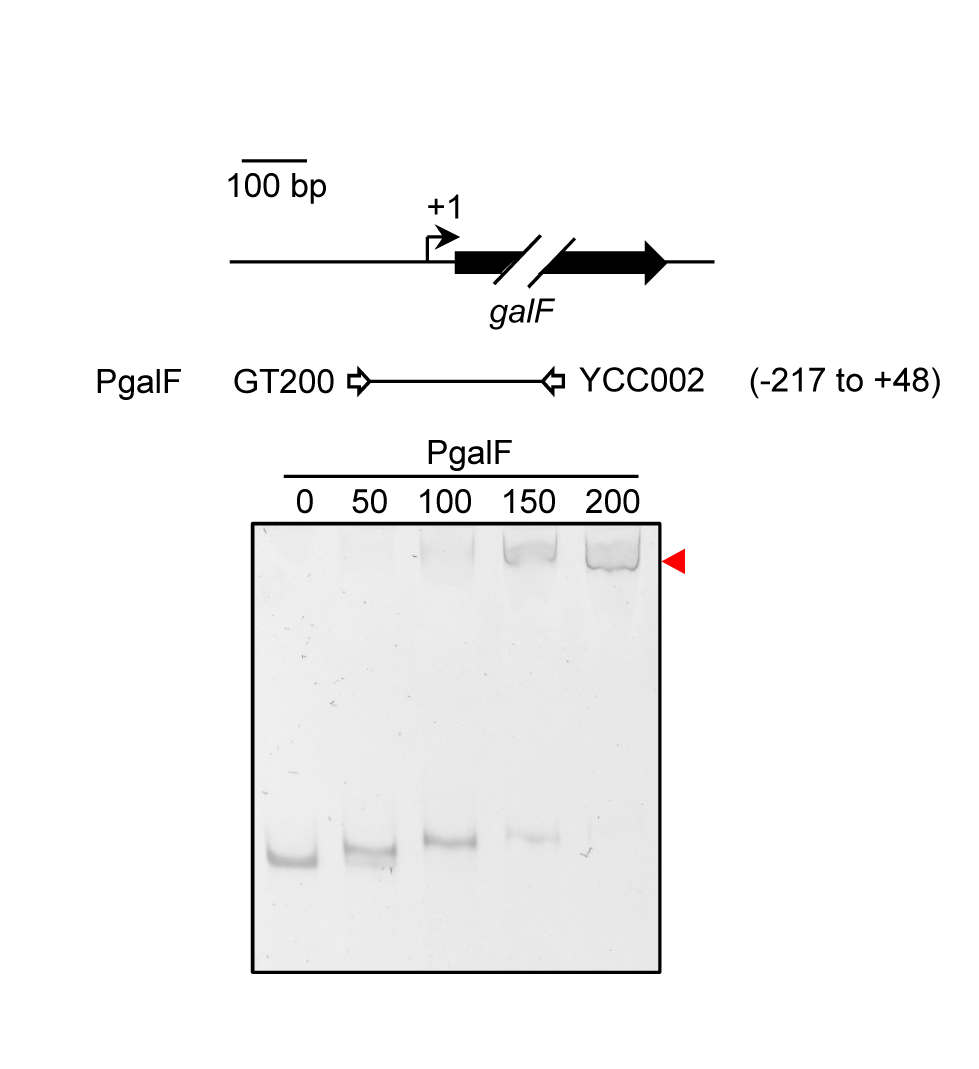

Supplement: Figure S2 — CRP directly binds to P galF . Diagrammatic representation of the galF loci. The large arrows represent the open reading frames. The relative positions of the primer set used in PCR-amplification of the DNA probes are indicated, and the numbers denote the positions relative to the translational start site. Name of the DNA probes are shown on the left. Different concentrations of purified His6-CRP were incubated with 10 ng of the upstream regions of galF. Following incubation at room temperature for 30 min, the mixtures were analyzed on a 5% non-denaturing polyacrylamide gel containing 200 µM cAMP. The gel was stained with SYBR Green I dye and photographed. (TIF) [file pone.0054430.s002.tif]

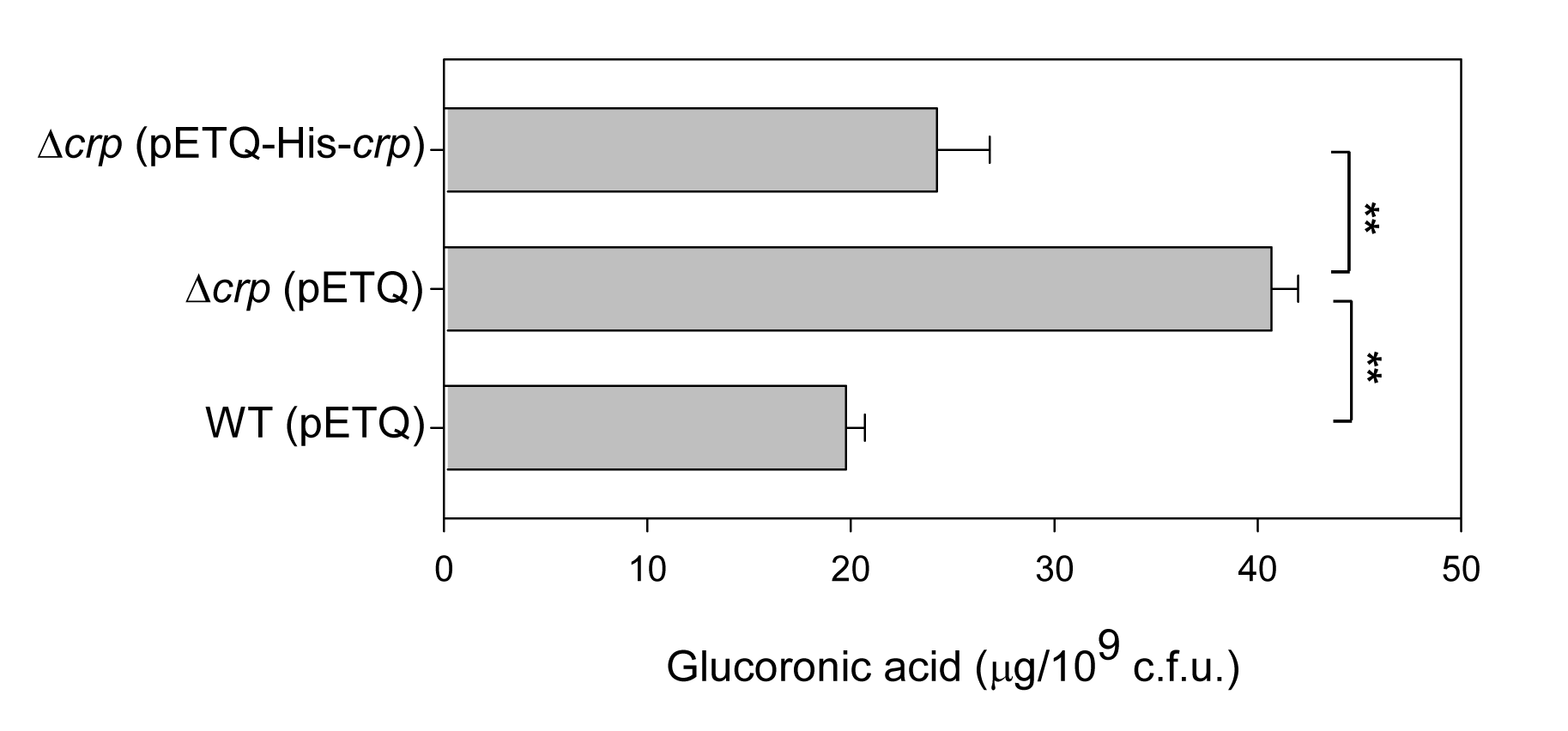

Supplement: Figure S3 — Induced expression of His6-CRP complements the effect of crp mutation on CPS biosynthesis. CPS levels of K. pneumoniae strains carrying the IPTG inducible vector pETQ or pETQ-His-crp, as shown in the left panel, were determined. Bacteria were grown in LB medium with 100 µM IPTG at 37°C with agitation. **P<0.01 compared to the indicated group. (TIF) [file pone.0054430.s003.tif]
